# Ten-year population-based assessment of multimorbidity burden progression in a regional cohort of 5.5 million adults

**DOI:** 10.1038/s41746-026-02395-x

**Published:** 2026-01-31

**Authors:** Damià Valero-Bover, David Monterde, Gerard Carot-Sans, Emili Vela, Rubèn González-Colom, Josep Roca, Caridad Pontes, Xabier Michelena, Maria Mercedes Nogueras, Pilar Aparicio, Inmaculada Corrales, Teresa Biec, Isaac Cano, Jordi Piera-Jiménez

**Affiliations:** 1 Servei Català de la Salut (CatSalut), Barcelona, Spain; 2 Digitalization for the Sustainability of the Healthcare System (DS3) Research Group, Barcelona, Spain; 3 Departament de Medicina, Universitat de Barcelona (UB), Barcelona, Spain; 4 Institut Català de la Salut (ICS), Barcelona, Spain; 5 Fundació de Recerca Clínic Barcelona – Institut d’Investigacions Biomèdiques August Pi i Sunyer (FRCB-IDIBAPS), Barcelona, Spain; 6 Servei de Farmacologia Clínica, Hospital de la Santa Creu i Sant Pau, Barcelona, Spain; 7 Departament de Farmacologia, de Terapéutica i de Toxicologia, Universitat Autònoma de Barcelona, Bellaterra, Spain; 8 Agència de Qualitat i Avaluació Sanitàries de Catalunya (AQuAS), Barcelona, Spain; 9 Subdirección General de Calidad Asistencial, Dirección General de Salud Pública y Equidad en Salud, Ministry of Health, Madrid, Spain; 10 Clinical Informatics Service, Hospital Clínic de Barcelona, Barcelona, Spain; 11 Faculty of Informatics, Multimedia and Telecommunications, Universitat Oberta de Catalunya, Barcelona, Spain

**Keywords:** Diseases, Health care, Medical research, Risk factors

## Abstract

Multimorbidity, a major driver of healthcare demand and clinical complexity, is often addressed in a disease-centric manner and remains insufficiently understood in its population-level dynamics. Using data from a 10-year population-based cohort of 5.5 million adults in Catalonia, Spain, we quantified multimorbidity-associated clinical complexity using the Adjusted Morbidity Groups (AMG) index to predict progression from low/moderate ( < P_80_) to high/very high ( ≥ P_80_) complexity. Machine learning models identified predictive factors, while network analyses explored co-occurrence patterns among chronic conditions. During follow-up, 39.2% of the individuals who remained alive throughout the analysis period transitioned to high/very high complexity. Baseline AMG score was the strongest predictor of progression, surpassing models relying solely on individual diagnoses. The most prevalent conditions were nutritional and endocrine disorders, anxiety, and hypertension, with notable sequential links between mental and physical disorders. Findings emphasize the need for integrated, patient-centred care strategies and population-based prevention approaches to mitigate multimorbidity progression.

## Introduction

Multimorbidity (i.e., the co-occurrence of more than one chronic condition in one individual) has become one of the most pressing challenges facing healthcare systems worldwide^1,2^. Despite its high prevalence, which is expected to rise with population ageing, and unhealthy lifestyles, healthcare systems continue to manage multimorbidity through a predominantly single disease-oriented rather than patient-centred approach, overlooking the complex interactions between conditions and their treatments^3,4^.

The use of multimorbidity quantifiers (also known as case-mix tools) has been proposed to comprehensively identify the needs of patients with multimorbidity^5,6^. With the increasing digitalization of healthcare systems, this approach not only provides clinicians with a more accurate and comprehensive population-based view of patients with multimorbidity but also enables characterizing patient’s health complexity in advance^7^. In recent years, this broader perspective has expanded to include the analysis of disease trajectories and networks, enhancing our understanding of multimorbidity^8–10^. Despite the potential of electronically available and structured healthcare data for comprehensive approaches to multimorbidity, the single-disease framework remains dominant in healthcare and medical research^11^.

In previous research, we took advantage of the comprehensive data collection from primary and specialized care in Catalonia (Spain)^7^ to develop the Adjusted Morbidity Groups (AMG), a summary measure of multimorbidity that allows for stratifying the entire population into meaningful risk groups based on their multimorbidity burden^12,13^. These multimorbidity-based risk groups showed strong correlation with health outcomes and resource utilization in different European populations, demonstrating their effectiveness in reflecting disease burden^13–16^.

Early identification of patients at risk of transitioning to high morbidity burden is essential for developing targeted public health strategies^7^, supporting secondary prevention in clinical practice, improving the proactive management of patients with multimorbidity, and fostering precision medicine. The aims of the present exercise were to better understand the pathways driving multimorbidity by means of identifying the factors that may predict the transition to high morbidity burden and exploring the clinical patterns of concurrence for the most prevalent chronic conditions.

## Results

### Study population and multimorbidity

The study cohort included 6,205,308 adults who were living in Catalonia at the beginning of the observation period (January 1, 2013). Of them, 5,538,657 (89.3%) remained alive after ten years, by the end of the observation period (December 31, 2022), and were therefore included in the primarily analysis. Of the remaining 666,651 individuals, 319,834 (5.2% over the entire cohort) experienced the event of interest for predictive modelling (i.e., transition from low/moderate to high/very high clinical complexity, based on AMG ≥ P_80_) (Fig. S1 in Supplementary Information file). Table 1 summarizes the main demographic and clinical characteristics of the primary study population at baseline and at the end of the study.

**Table 1:** Main demographic and clinical characteristics of the study cohort at baseline and end of study period

	Baseline (*N* = 5,538,657)	End of study period (*N* = 5,538,657)
Age groups, *n (%)*		
18-40	2,628,945 (47.5%)	1,321,118 (23.9%)
40-60	1,941,034 (35.1%)	2,416,500 (43.6%)
60-70	605,763 (10.9%)	832,361 (15%)
70-80	300,189 (5.4%)	605,763 (10.9%)
>80	62,726 (1.1%)	362,915 (6.6%)
Age (years), *mean (SD)*	43.67 (16.1)	53.67 (16.1)
Sex (Female), *n (%)*	2,867,086 (51.8%)	2,867,086 (51.8%)
Multimorbidity strata^a^, *n (%)*		
Low risk ( < P_50_)	4,201,204 (75.85%)	2,769,406 (50%)
Moderate risk ( ≥ P_50_ to <P_80_)	1,112,213 (20.08%)	1,661,597 (30%)
High risk ( ≥ P_80_ to <P_95_)	212,521 (3.84%)	830,747 (15%)
Very-high risk ( ≥ P_95_)	12,719 (0.23%)	276,907 (5%)
Multimorbidity burden (AMG index) ^a^		
AMG index, *mean (SD) [IQR]*	1.62 (2.4) [0, 2.4]	4.15 (4.94) [0.4, 6]
AMG index at previous disease, *mean (SD)*	1.06 (2.1)	3.3 (4.6)
AMG index at first disease, *mean (SD)*	0.57 (0.7)	0.79 (0.6)
Time since onset of previous disease (years)^b^, *mean (SD)*	3.75 (3.4)	2.68 (3.6)
Time since onset of first disease (years)^b^, *mean (SD)*	7.47 (3.9)	8.96 (7.4)
Number of chronic conditions, *n (%)*		
0	2,473,021 (44.7%)	1,170,750 (21.1%)
1	1,041,140 (18.8%)	791,211 (14.3%)
2	710,381 (12.8%)	685,692 (12.4%)
3	480,795 (8.7%)	576,905 (10.4%)
4	318,380 (5.7%)	477,132 (8.6%)
5	205,889 (3.7%)	391,647 (7.1%)
6	129,019 (2.3%)	319,431 (5.8%)
7	77,970 (1.4%)	258,797 (4.7%)
≥8	102,062 (1.8%)	867,092 (15.7%)

*AMG* Adjusted Morbidity Group, *IQR* interquartile range (25th and 75th percentiles), *SD* standard deviation.

^a^The threshold for high/very-high risk correspond to values of the AMG index above the 80th percentile. ^b^ Estimated considering only individuals with at least one condition at each of the time points: baseline (*N* = 3,065,636) and end of study period (*N* = 4,367,907).

**Fig. 1:**
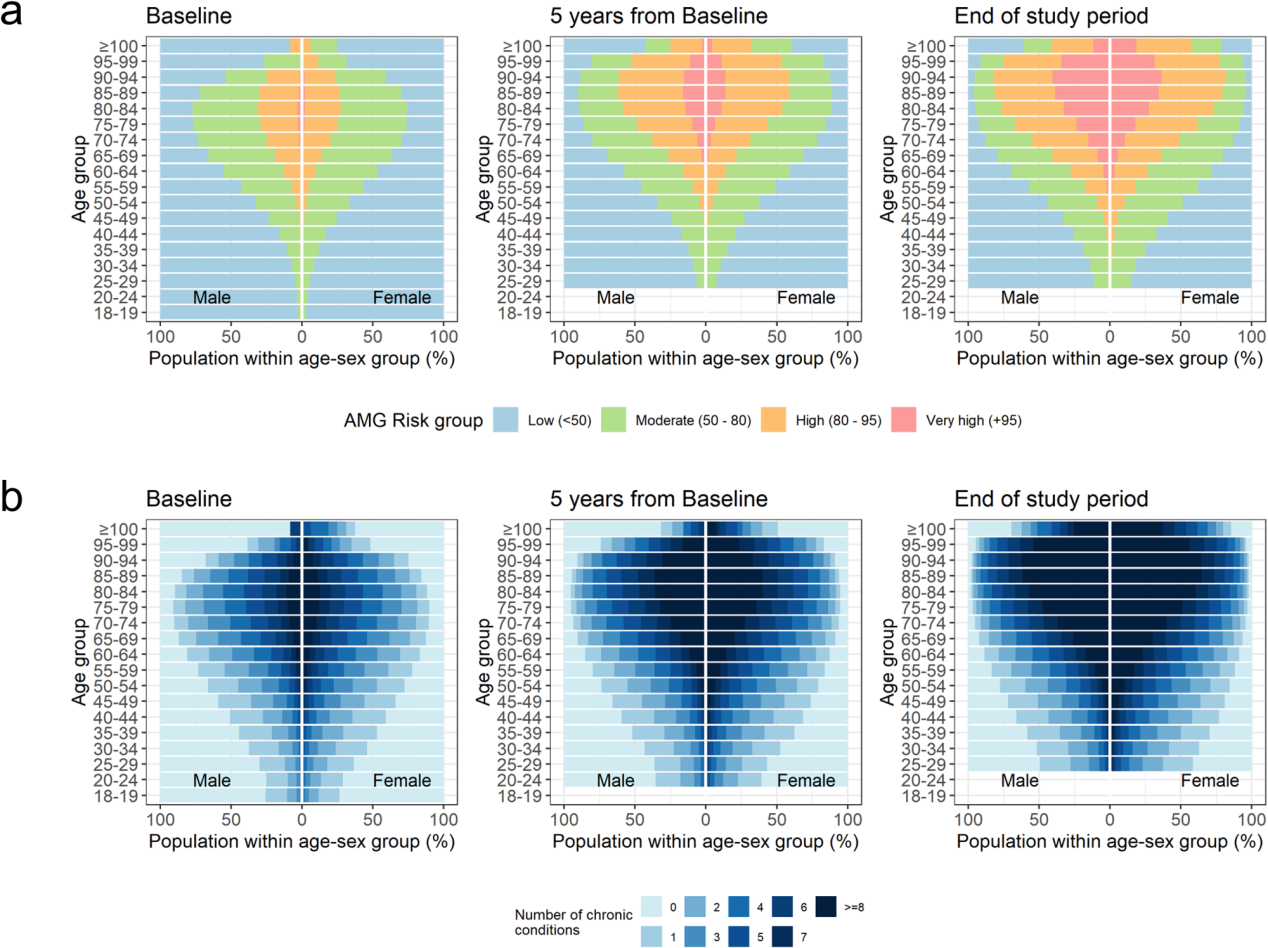
Progression of comorbidity burden throughout the study period across age groups and sex. **a** Risk groups of the Adjusted Morbidity Groups. **b** Rough number of comorbid conditions.

Throughout the ten-year follow-up, the median number of chronic conditions per individual increased from 1 (IQR 0, 2) to 3 (1, 6) (Fig. 1a), being more pronounced in the upper age groups. Up to 2,173,767 (39.2%) individuals experienced a transition towards a higher multimorbidity strata, with 882,414 (15.9%) moving from the low/moderate risk groups ( < P_80_) to the high/very-high risk groups ( ≥ P_80_) (Figure S2) Among those classified as low risk at baseline ( < P_50_), 65.9% remained stable throughout follow-up, compared to 44.4% in the moderate-risk group ( ≥ P_50_ to <P_80_) and 41.6% in the high-risk group ( ≥ P_80_ to <P_95_).

Figure 1B illustrates the progress of morbidity burden by age. No gender differences were observed in the increase of morbidity with age. Overall, the most prevalent conditions at the end of the study period were nutritional and endocrine disorders ―mostly obesity and lipid disorders (metabolic syndrome)― (25.3%), anxiety disorders ―mostly unspecified anxiety disorders and other mixed anxiety disorders― (24.3%), and essential hypertension (23.5%) (Table S1 in Supplementary Information file).

**Fig. 2:**
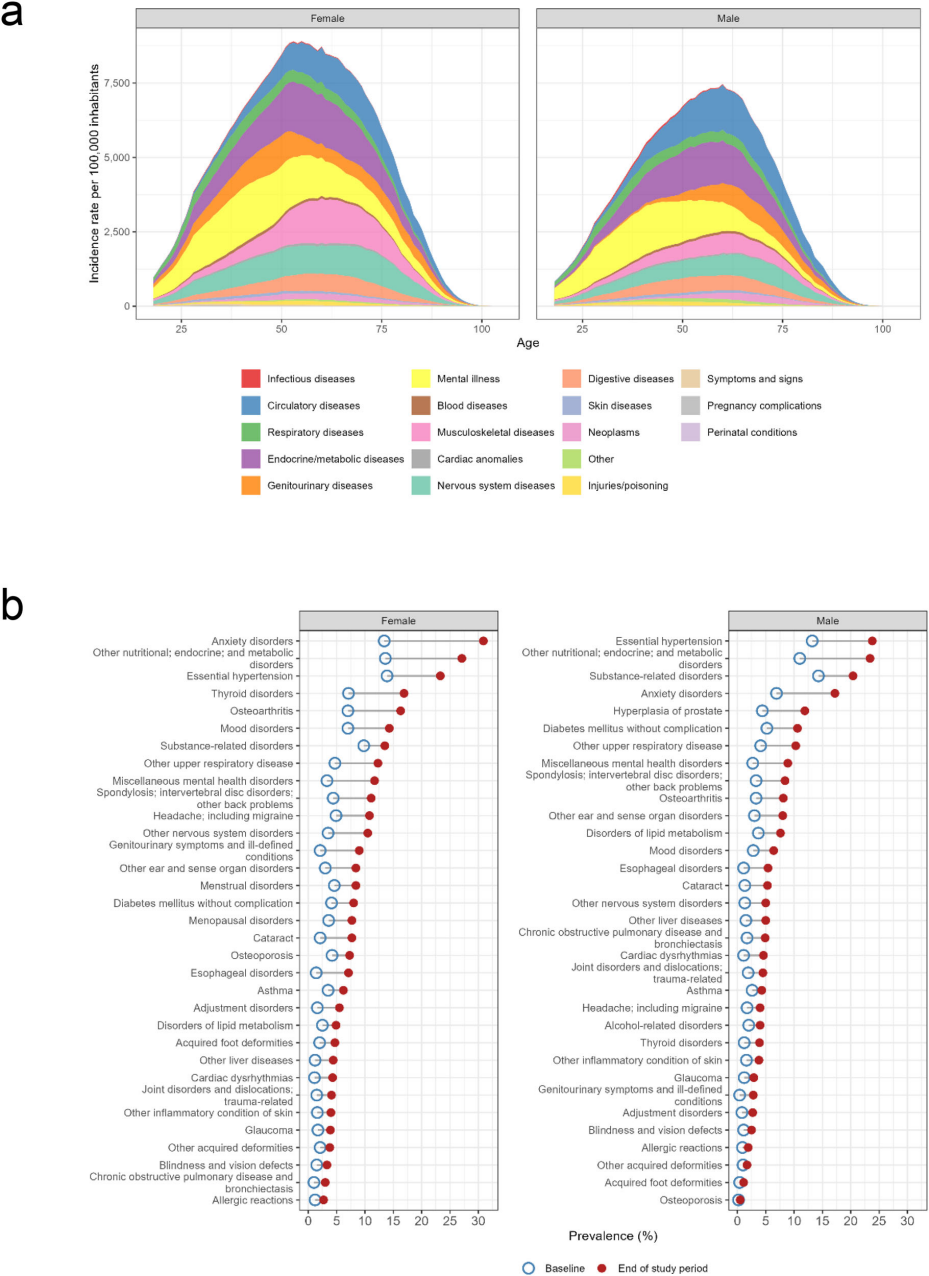
Chronic conditions in the study population. **a** Onset of chronic conditions (groups of the Clinical Classification Software for chronic diseases), according to sex and age at diagnosis. **b** Prevalence of comorbidities at the beginning and end of the study period (only comorbidities with prevalence >1% at the end of the study period are shown). The corresponding prevalence of ICD-10-CM codes are shown in Table S1 (Supplementary Information file).

Figure 2a summarizes the onset of new chronic conditions at each age group among individuals with at least one chronic condition during the study period. In both males and females, the onset of mental illness was remarkably higher in young adults and decreased with age. Conversely, the onset of diseases of the circulatory system, more frequent among men, increased with age. Diseases of the respiratory system showed a relatively stable onset across the life course. Among women, the onset of diseases of the genitourinary system occurred at all ages, whereas in men, it became pervasive after 50 years. Women also showed higher percentages of musculoskeletal disorders than men, with a particularly higher onset rate at ages above 50 years.

The conditions with greater increase in prevalence among men were nutritional, endocrine, and metabolic disorders (from 11.0% at baseline to 23.4% at the end of the investigation period), essential hypertension (13.2% to 23.8%), and anxiety disorders (6.9% to 17.2%) (Fig. 2b). The leading conditions in terms of prevalence increase among women were anxiety disorders (13.4% to 30.9%), nutritional, endocrine, and metabolic disorders (13.6% to 27.1%) and thyroid disorders (7.1% to 16.9%). The three conditions that most frequently co-occurred with transition from low/moderate to high/very high clinical risk (AMG ≥ P_80_) were chronic kidney disease, hypertension, and osteoarthritis (Fig. S3).

### Models for multimorbidity progression

**Fig. 3:**
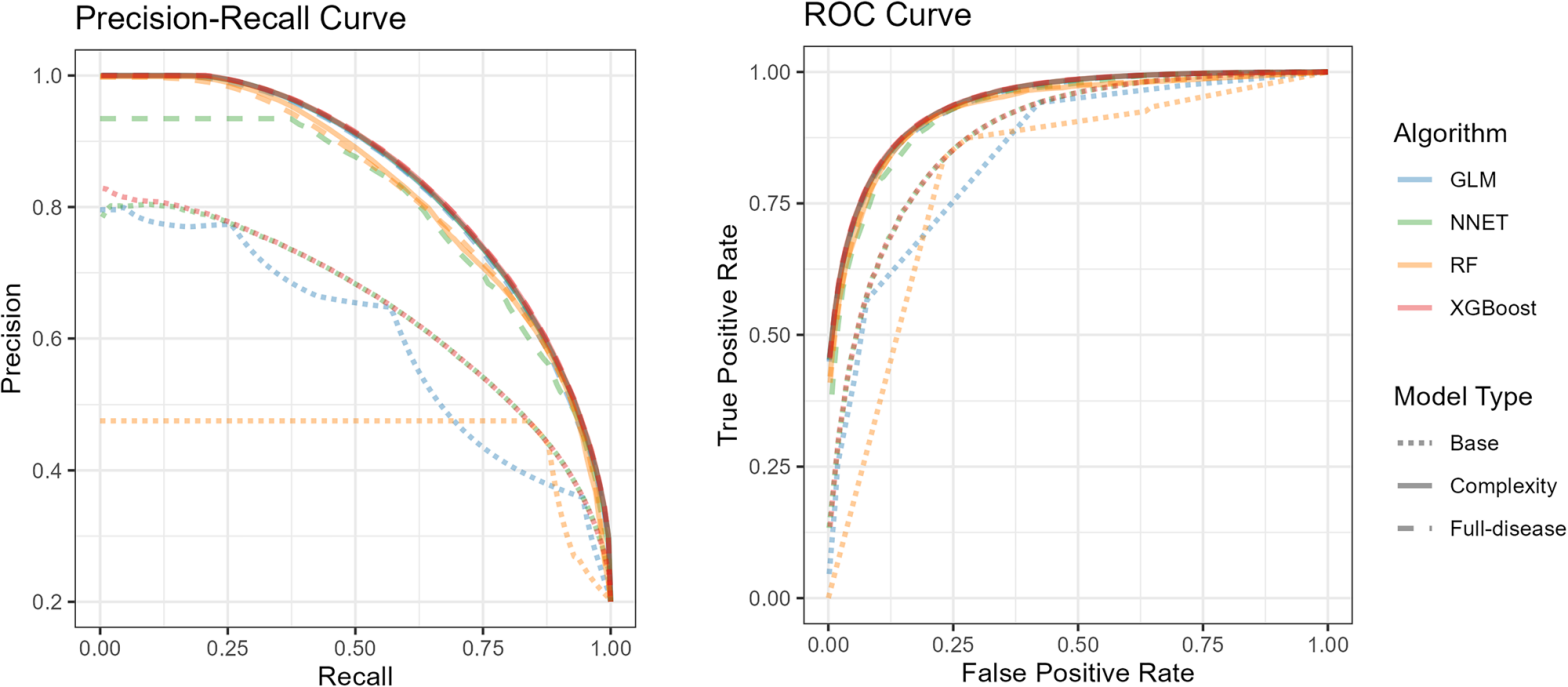
Performance of the models for predicting transition to high/very-high clinical risk (Adjusted Morbidity Groups [AMG] index ≥P_80_). Precision-recall (PR) and receiver operating characteristics (ROC) curves of the base (age and sex) and complexity (including AMG index at different stages) models, replicated for the following architectures: generalized-linear model (GLM), neural network (NNET), random forest (RF), and X-gradient boost (XGBoost).

Transitions from low/moderate to high/very high clinical risk (AMG ≥ P_80_) could be predicted with moderate to good discrimination across the different modelling approaches (Table S2). The predictive performance improved consistently when adding morbidity indicators beyond basic demographic variables (Fig. 3). Models that included longitudinal multimorbidity information—such as AMG scores and timing of diagnoses—substantially outperformed models that relied solely on age and sex. Table S3 summarizes the relative contribution of the most relevant variables to explaining the increase in clinical complexity. Multimorbidity burden (measured with the AMG index) was among the two leading variables contributing to complexity increase in all models; age (for RF model) and number of chronic conditions (for NET model) were also relevant contributors to the outcome (Table S3). Dedicated models developed for subgroups of individuals with specific chronic conditions showed no significant performance improvement compared to the general model applied to the same subgroups (Fig. S4). This trend was similar for the four predictive modelling approaches tested in the study (Table S2).

The analysis of disease co-occurrence directed networks revealed structured patterns in the development of multimorbidity across the population, providing further insight into the temporal dynamics of these associations. Although 16,157 diagnostic pairs were identified, only a small subset of them showed a relevant prevalence in the population, while most pairs (86.3%; 13,941/16,157) were present in less than 0.1% (*n* < 5000) of the population, featuring the highly heterogeneous nature of chronic disease clustering (Fig. S5). The frequency of the most prevalent diagnosis pairs and triads are summarized in Table S4 and Table S5, respectively.

### Networks of chronic conditions

**Fig. 4:**
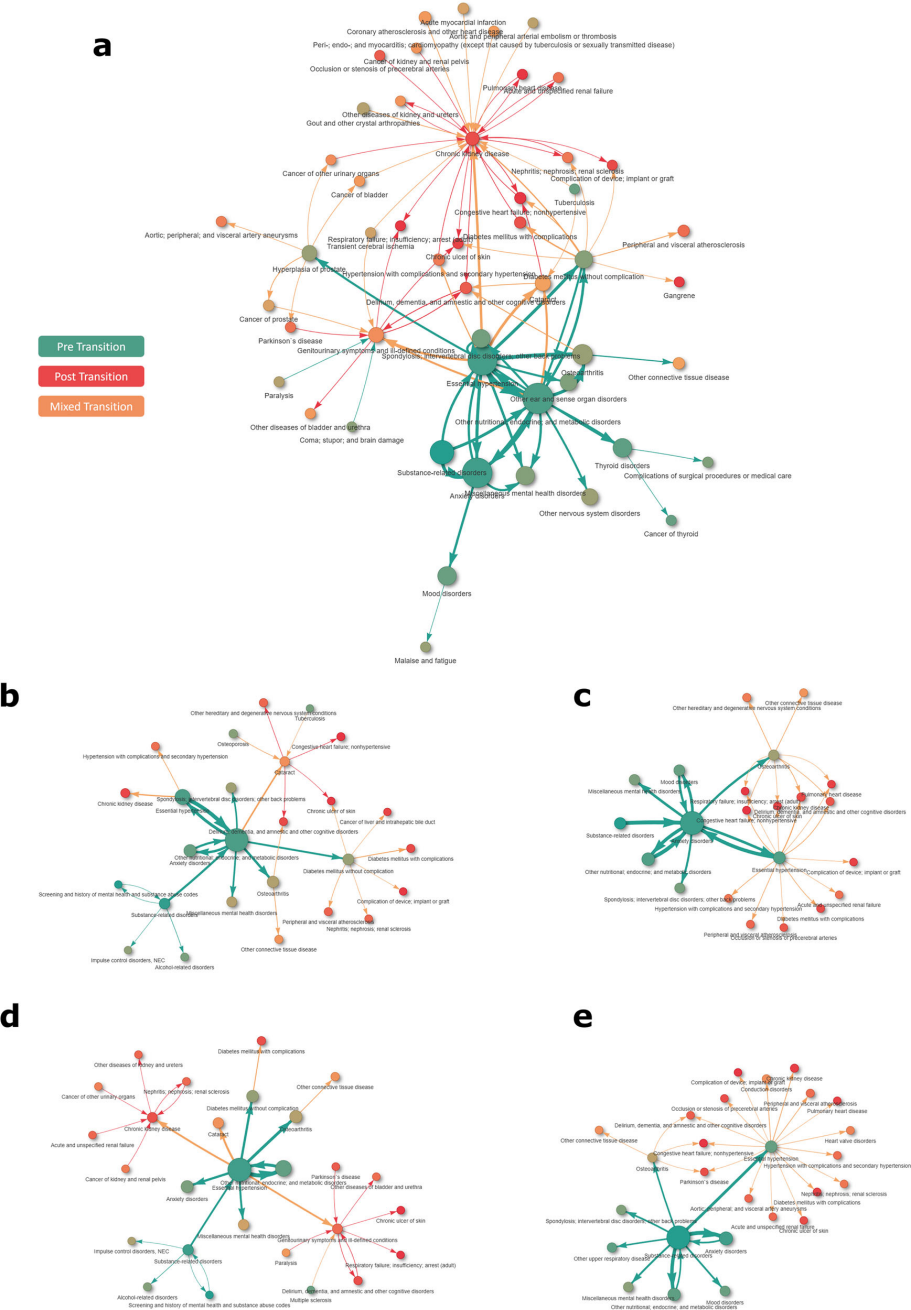
General population network and disease-centred networks for chronic conditions with leading prevalence. **a** General population network - thirty leading interactions and secondary connections including the top 60 risk ratios. Colors indicate occurrence before (green) or after (red) transition towards high/very-high risk (Adjusted Morbidity Groups index ≥P_80_); orange indicates mixed transition or condition onset. The area of the circles is proportional to the prevalence of the condition at the end of the study period; line width is proportional to number of connections, normalized to a 1-10 scale. **b** Disease-centred networks - Other nutritional; endocrine; and metabolic disorders. **c** Disease-centred networks - Anxiety Disorders. **d** Disease-centred networks - Essential hypertension. **e** Disease-centred networks - Substance-related disorders. The disease-centred networks can be explored in the following dynamic html platform: https://ds3-siscat.github.io/multimorbidity-progression/.

Figure 4a shows the overall disease network, including the most prevalent and relevant interactions. Briefly, the combination of essential hypertension with nutritional, endocrine, and metabolic disorders was the most prevalent, affecting 687,329 individuals (12.4%). Other high-frequency co-occurrences included combinations of these same conditions with osteoarthritis, anxiety disorders, substance-related disorders, or diabetes mellitus without complications. The overall structure of the network shows a temporal shift from early contributions of mental health and metabolic conditions towards later cardiovascular and renal complications, reflecting the dynamic layering of chronic disease burden.

The detailed networks for the four chronic conditions with the leading prevalence at the end of the study period are summarized in Fig. 4b–e; the corresponding networks for the 20 most prevalent conditions are available from the supplementary dynamic html page at https://ds3-siscat.github.io/multimorbidity-progression/. Fig. S6 and Fig. S7 show the general and four leading dedicated networks, respectively, for men and women separately. Nutritional and endocrine disorders, primarily obesity and lipid disorders, were frequently followed by the development of essential hypertension, type 2 diabetes, osteoarthritis, and several mental health disorders (Fig. 4b). These patterns were predominantly observed before individuals transitioned to a high-risk status ( ≥ P_80_).

Anxiety disorders, often preceded by substance-related disorders (mainly tobacco use), were associated with a broad set of chronic conditions, including both physical and neuropsychiatric disorders (Fig. 4c). These associations mostly occurred pre-transition ( < P_80_), pointing to anxiety as a potential early indicator of future multimorbidity. Conversely, essential hypertension (Fig. 4d) was more often associated with conditions that emerged after the risk transition ( ≥ P_80_), including osteoarthritis, various endocrine disorders, mental health conditions, chronic kidney disease, and genitourinary symptoms.

Substance-related disorders were associated with increased risk for a range of subsequent conditions, notably anxiety, mood disorders, essential hypertension, and nutritional and endocrine disorders (Fig. 4e). Most of these associations were also observed before the transition to high clinical risk ( < P_80_).

Most disease clusters were broadly shared between men and women; however, some sex-specific patterns emerged (Fig. S7 for general network for female and male and Fig. S8 for the sex-specific, dedicated network). Among men, prostate hyperplasia was frequently preceded by metabolic disorders, anxiety, and substance-related conditions. In contrast, among women, metabolic disorders and hypertension commonly preceded the onset of genitourinary symptoms in the context of the complexity transition.

## Discussion

Our retrospective analysis of multimorbidity progression over 10 years in a population-based cohort of 5.5 million adults confirmed the high prevalence of multimorbidity and its cumulative nature, particularly in older age groups, underscoring important implications at the societal and healthcare system level. The assessment of transition dynamics towards higher risk multimorbidity strata revealed that such shifts are not only frequent but also exhibit predictable patterns.

The modelling of morbidity progression provided two relevant lessons with practical implications in the public health and in the clinical scenarios. Firstly, the baseline morbidity burden was the most important contributor explaining multimorbidity progression across health risk strata, well beyond other covariates such as age, gender and/or single-disease approaches. Secondly, the outcomes were highly consistent across different families of predictive modelling approaches, supporting the robustness of the study results. Together, both facts increase the understanding of the risks associated to morbidity burden and support the use of morbidity quantifiers in healthcare management and clinical practice.

Complementary to the longitudinal analysis, our network analysis identified relevant disease associations and their sequential appearance. Although not intended to establish causal relationships, these temporal sequences may act as useful indicators of clusters of conditions with shared determinants and healthcare needs, or signal subgroups in which the accumulation of specific co-occurring conditions is likely. As expected, our network analysis highlighted associations such as nutritional/metabolic disorders (mostly obesity) and essential hypertension, likely reflecting the clustering of metabolic and vascular risk factors commonly understood as components of the metabolic syndrome^17^. However, we also found that conditions often perceived as low-priority or manageable in isolation, such as anxiety-depression, act as early precedents of clinical deterioration in cardiovascular diseases (e.g., hypertension) and other physical chronic conditions. These findings are consistent with previous developments of risk scores for chronic mental health conditions, which highlighted the strong relationship between mental health and multimorbidity burden, including physical conditions^18,19^, and emphasise the importance of their early management for long-term prevention of more severe conditions. However, the implementation of tailored, community-based interventions for individuals at risk of developing specific morbidity clusters remains a challenge. Further research is needed to elucidate the underlying mechanisms of clustering and to design effective, evidence-based interventional strategies^19–21^. The interactive HTML module developed for network exploration offers a valuable platform for future investigations into the causal mechanisms explaining the observed clusters and sequences.

Taken together, the study results stress the need for adopting a more comprehensive approach towards preventive management of multimorbidity in terms of health policies and patient-centred care. Predictive modelling could facilitate the forecasting of demand for primary care, emergency services, and specialist professionals, thereby enabling more efficient workforce and budget planning. The practical value of multimorbidity-based risk assessment for resource allocation was evident during the COVID-19 crisis, when this approach enabled vaccine prioritisation based on predicted risk of hospitalisation or severe disease rather than on crude surrogates such as age^22^. From a public health perspective, multimorbidity-driven, population-based risk assessment offers substantial advantages by enabling the stratification of populations according to their susceptibility to rapid deterioration and the early identification of high-risk individuals. This facilitates the design and implementation of tailored, community-level preventive interventions that respond to the actual constellation of risks present in each subgroup. Our multimorbidity networks align with cumulative evidence that shows how many risk factors overlap (e.g., tobacco and alcohol use clustering with anxiety, metabolic, cardiovascular, and respiratory diseases), suggesting that preventive strategies can be shared across conditions and evaluated through multidisciplinary monitoring frameworks. In our approach to multimorbidity measurement, the information needed to flag individuals at elevated risk is already embedded within routine electronic health records; hence, these assessments can be conducted without additional clinical workload, allowing healthcare systems to efficiently target interventions where they are most needed. This approach enhances equity, aligns with integrated, person-centred care models, and offers a pragmatic pathway to reducing preventable clinical deterioration and its associated economic impact.

In the clinical domain, identifying patients at higher risk of escalating multimorbidity burden presents a critical opportunity to prioritise targeted prevention strategies, particularly in resource-limited settings. Current secondary prevention approaches remain predominantly diagnosis-centred, focusing on the progression of individual conditions or the emergence of secondary diseases traditionally classified as complications of a primary diagnosis. However, our findings align with growing evidence highlighting the complex interplay between diseases, wherein risk extends beyond narrowly defined complications to encompass conditions across distinct systems or domains^21,23–26^. In this regard, recent experiences illustrate how comprehensive and data-driven multimorbidity measurement (such as that proposed in our model) can play a key role in programs aimed at preventing early readmissions after hospital discharge^27^, or in the ongoing evaluation of community-based hybrid care interventions designed to reduce potentially avoidable hospitalizations among high-risk patients with COPD or severe asthma^28,29^.

These observations, along with the demonstration that the baseline multimorbidity burden (rather than specific conditions alone) is the strongest predictor of rising health complexity, highlight the need for transitioning from a single-disease framework (which often results in fragmented care) to a comprehensive, population-health-based model that allows a truly patient-centred framework addressing complexity increase aside from disease progression. In this regard, a model informing about complexity increase as a whole can support integrated preventative interventions^11^.

The importance of multimorbidity as a major determinant of population health (and its potential to inform healthcare planning and policy) has been highlighted by several authors^30,31^, including those demonstrating that multimorbidity prevalence can anticipate healthcare resource needs^32–35^. Our descriptive findings align with this body of work, confirming not only the substantial prevalence of comorbidities in the general population but also the specific conditions that most strongly drive disease burden. However, much of the prior literature has focused on healthcare costs and has typically relied on static assessments that do not capture how individuals transition across multimorbidity or complexity levels over time. Regarding the attempt to cluster chronic conditions into disease networks, previous studies have tended to either provide high-level descriptions of disease clustering from an epidemiological perspective^10,36–38^ or develop trajectory-based analyses aimed at predicting the next likely condition^8^. In contrast, our approach predicts transitions from low to high multimorbidity-associated complexity level within a 10-year window based on cumulative diagnoses recorded in electronic health records. Although categorising clinical complexity into a limited number of strata inevitably reduces granularity, this simplification makes the approach more practical for routine use by healthcare organisations across the range of applications described above. Our study is strengthened by three relevant features. First, the regional population-health approach for the ten-year retrospective follow-up (2013–2022), including historical patients’ data since early 2000s^7^. Second, the use of the AMG as morbidity quantifier should be viewed as a factor facilitating the applicability of multimorbidity predictors due to its proven transferability across EU countries^5,6,14–16^. Finally, the study design combining population-based predictive modelling of multimorbidity progression and network analysis of disease trajectories and associations (available as dynamic html for exploration) may open novel windows of opportunity to address current multimorbidity challenges.

However, our findings should be interpreted considering some inherent limitations of the study design. First, we defined reaching high/very-high risk ( ≥ P_80_) over a 10-year period as the primary outcome to explore factors influencing the worsening of clinical complexity, excluding deaths. While this approach is suitable for long-term projections, alternative metrics, such as the rate of increase in the multimorbidity index or transitions between the four risk strata might be suitable for chronic disorders with rapid evolution. Furthermore, individuals with the most aggressive disease trajectories or the highest initial risk may have died earlier and thus were excluded from the analysis. Although individuals experiencing the endpoint transition and dying during the study period accounted for only 5% of the population, this introduces an immortal time–related bias that likely underestimates the overall burden and pace of multimorbidity progression in the general population. Nevertheless, from a healthcare services and public health perspective, focusing on trajectories in individuals with slower, and potentially preventable, progression remains meaningful.

A second limitation was that, regardless of the metric used, our analysis was constrained by the retrospective nature of the routinely collected care data, which lack critical information on factors such as health behaviours and social stressors. Although these variables are frequently overlooked in studies of disease progression, they may play a significant role in the escalation of clinical complexity over time. Nonetheless, the exclusive use of routine care data enhances the feasibility of implementing our models, which shall be confirmed through external validations, in real-world settings, thereby supporting their potential to inform decision-making in practice. In this line, the analysis of temporal disease trajectories in the current research uncovered our still limited knowledge on the underlying mechanisms of multimorbidity progression, which was beyond our current research objectives. Finally, although a cross-validation strategy with train-test split of the population was used for model development and assessment, all analyses have been conducted on a given population under the same country and healthcare system. Future studies shall investigate the external validity of our conclusions by testing the models in different populations.

In summary, the findings of this study advocate for a paradigm shift from single-disease management to integrated, patient-centred approaches that address multimorbidity holistically. The results highlight the utility of multimorbidity-based models for public health planning, enabling more accurate forecasting of healthcare needs, targeted preventive interventions for susceptible risk groups, and development of context-specific care algorithms. Such approaches could significantly improve resource allocation and improve clinical practice while addressing the complex interplay between physical and mental health conditions observed in the study.

## Methods

### Study design and population

This retrospective analysis examined a full-population cohort of adult residents in Catalonia, a Northeastern Spanish region with a population of 8 million. The study included all adults (i.e., individuals aged 18 years or older) living in the region as of January 01, 2013. The primary analysis population consisted of all individuals who remained alive at the end of the observation period on December 31, 2022. For individuals dying within the analysis period, a sensitivity analysis was conducted to assess the potential impact of immortal bias. Health data available for the study cohort before the follow-up period (2013) were also included for predictive modelling purposes. Additionally, we mapped networks of concomitant chronic conditions within the cohort. Figure S8 illustrates the overall study design.

All data were retrieved from the Catalan Health Surveillance System (CHSS), a central database of the Catalan Health Service designed for public health purposes^7^. The Catalan Health Service provides universal public primary and specialized care to the entire population of Catalonia through a network of 64 general hospitals, 27 psychiatry hospitals, 375 primary care centres, 91 skilled nursing facilities for intermediate care, and 130 ambulatory mental health facilities. The CHSS systematically collects relevant data regarding the demographic and clinical characteristics of all insured citizens, as well as healthcare resource utilization, including hospitalizations, visits to the emergency room, and visits to primary care, among others. All records were linked through unique patient identifiers before data extraction by personnel unrelated to the investigator team and fully anonymised in the database released for analysis.

The study protocol was approved by the Ethics Committee of the Hospital Clínic de Barcelona (Spain) (ref. HCB/2020/1051), which waived the collection of informed consent for the secondary use of anonymized healthcare data. Although the study did not involve experimental procedures, the applicable principles of the Declaration of Helsinki for research involving human beings were followed. The manuscript for results reporting has been prepared according to the guidelines for transparent reporting of multivariable prediction models for individual prognosis or diagnosis (TRIPOD)^39^; the corresponding checklist is provided in the Supplementary Information file.

### Predictive modelling of multimorbidity progression

The primary outcome of the predictive models was the progression towards high/very high multimorbidity burden, measured using the AMG index. The AMG index is a numerical measure that estimates the clinical complexity of a patient based on the weighted sum of all possible chronic conditions and recent acute conditions considering all possible diagnostic groups.

The methodological details of index development and validation are reported elsewhere^12,16,40^. Figure S9 illustrates the operation algorithm of the AMG. Briefly, the AMG system estimates an individual’s clinical complexity by processing all recorded diagnoses together with basic personal information (identifier, age, and sex), using diagnostic data coded in CD-9-CM, ICD-10, ICD-10-CM, or ICPC-1/2. Before constructing the index, the algorithm validates diagnoses to ensure consistency with age, sex, and the analysis period, discarding incompatible or invalid entries. Diagnoses are then grouped into standardized diagnostic code groups based on the Clinical Classification Software^41^, and each diagnostic group is labelled as acute (up to one year prior to index estimate), chronic, neoplastic, or pregnancy-related according to the healthcare cost and utilization project (HCUP) criteria for chronicity. Chronic conditions are additionally mapped to organ systems. For every diagnostic group, the AMG assigns a complexity weight informed by empirical modelling of mortality, healthcare utilisation (scheduled and unscheduled hospitalizations, primary care contacts), and medication burden in the reference population of Catalonia. These weighted values are summed at the individual level to generate a continuous morbidity index, which reflects both the number and severity of coexisting conditions. The algorithm subsequently allocates individuals to mutually exclusive morbidity groups following a hierarchical structure that prioritizes active neoplasia, pregnancy/childbirth, and the number of organ systems affected by chronic conditions, with additional consideration of acute illness and a final category for those classified as healthy.

Based on health outcomes and healthcare resource use, the entire population of Catalonia can be distributed across four AMG risk strata^5,12^: low risk (healthy stage, including AMG scores up to the 50th percentile of the total population, < P_50_), moderate risk (50th to 80th percentiles, ≥P_50_ to <P_80_), high risk (80th to 95th percentiles, ≥P_80_ to <P_95_), very high risk ( ≥ 95^th^ percentile, ≥P_95_).

Models were developed for the primary analysis population to predict the transition from low/moderate risk ( < P_80_) to high/very-high risk ( ≥ P_80_). Two types of analyses were conducted. First, three model specifications were built for the entire population: (1) a base model including age and sex; (2) a complexity model, which sequentially added the following predictors to the base model: number of chronic conditions, years since the first diagnosed chronic condition, years since the most recent chronic condition, the AMG index, the AMG index at the time of the first diagnosed chronic condition, and the AMG index at the time of the penultimate diagnosed chronic condition; and (3) a full-disease model that further included the presence or absence of each chronic condition as binary predictors. All models were replicated using four analytical approaches: generalized linear model (GLM), random forest, neural network, and extreme gradient boosting (XGBoost). Model performance was evaluated using the area under the receiver operating characteristics (AUROC) and the area under the precision-recall curve (AUPRC). All models were trained using cross-validation (70/30 train/test split with repeated 5-fold internal resampling). Performance metrics reported in the manuscript correspond exclusively to the held-out test sets, whereas cross-validation (repeated 5-fold resampling) was used only within the training partition to optimise model tuning and prevent overfitting.

Finally, the best-performing model from the population-wide analysis was compared against disease-specific dedicated models, which were trained on subsets of individuals with a given chronic condition at baseline (restricted to conditions with prevalence >1% at baseline). This allowed the evaluation of whether models tailored to subpopulations with specific baseline diseases differed from the general model when applied to the same subgroups. For individuals who died within the study period, a sensitivity analysis of progression across the stages of low/moderate risk ( < P_80_), high/very-high risk ( ≥ P_80_), and dead was conducted to ascertain the potential impact of immortal time-related bias on the findings.

### Network analysis of concomitant chronic conditions

Two complementary sets of directed multimorbidity networks were constructed using temporally ordered, patient-level diagnostic histories, where edges represented the observed sequence of appearance of each condition. To maintain clinical interpretability and reduce complexity, a maximum number of connection was established using a hierarchical strategy. For the first network (i.e., the general population network), we first selected the 30 most frequent directed pairs (i.e., sequential pairs of conditions, treating each possible direction independently for a given pair of conditions) across the whole cohort at the end of the study based on the number of individuals with each directed pair. The set of diseases involved in these primary connections was then used to identify the 60 next most relevant connections by co-occurrence risk ratio (Eq. 1), restricted to pairs that involved at least one of the primary diseases.

1 $${{RR}}_{\left\{{AB}\right\}}=\frac{O}{E}=\frac{{N}_{{ABC}}}{({N}_{A}\cdot {N}_{B})/N}=\frac{{N}_{{AB}}{\rm{\cdot }}N}{{N}_{A}\cdot {N}_{B}}$$

where:

- N_AB_ (O): The observed number of patients with both diseases A and B.
- N: The total number of patients.
- N_A_, N_B_: The number of patients with disease A and disease B, respectively.

The second network consisted of 20 disease-centred subnetworks, each focused on one of the most prevalent chronic conditions in the cohort. In each subnetwork, we first selected the ten most prevalent co-occurring conditions involving the central condition (i.e., secondary conditions). From these, we derived all possible directed pairs involving the secondary conditions and any other chronic condition, excluding the central condition itself, and selected the 20 with the highest adjusted risk ratios (tertiary conditions). Equation 2 shows the risk ratio estimate for triplets.

2 $${{RR}}_{\left\{{ABC}\right\}}=\frac{O}{E}\,=\,\frac{{N}_{{ABC}}}{({N}_{A}\cdot {N}_{B}\cdot {N}_{C})/{N}^{2}}=\frac{{N}_{{ABC}}{\rm{\cdot }}{N}^{2}}{{N}_{A}\cdot {N}_{B}\cdot {N}_{C}}$$

where:

- N_ABC_ (O): The observed number of patients who have all three diseases A, B, and C.
- N: The total number of patients.
- N_A_, N_B_, N_C_: The number of patients with disease A, disease B, and disease C, respectively.

For both the general population network and the disease-centred networks, we included all individuals diagnosed with the relevant conditions at any point in their longitudinal records, including diagnoses recorded before the study baseline. Directionality was determined independently for each pair. Pairs were further classified relative to the first transition to AMG ≥ P_80_ (pre-transition, post-transition, or mixed) and filtered to retain only those with more than 50 individuals and *p*-values below the Bonferroni-adjusted threshold, defined as follows: for *m* tested pairs, the significance threshold was set at α/m, where α = 0·05 and *m* was the total number of evaluated pairs. Adjusted p-values were obtained using the Bonferroni method implemented in R, and only pairs with *p* < α/m and involving ≥50 individuals were retained for network construction. Beyond statistical filtering, each condition pair was subsequently characterised by examining all instances in which both conditions appeared sequentially within an individual’s trajectory and determining whether these sequences occurred entirely before the transition to high/very-high risk, entirely after it, or spanned the transition. This approach allowed us to distinguish pairs whose temporal dynamics consistently preceded the onset of high complexity from those occurring predominantly in already high-risk individuals or those showing heterogeneous patterns across trajectories. Directed relationships were always treated as independent events, such that both A → B and B → A could be retained when each direction met the relevance criteria, reflecting the bidirectional relevance that can arise from distinct temporal pathways across the population.

Figures in the main document illustrate the overall population network, along with examples of the two primary disease-centred subnetworks. In addition, we developed an interactive HTML page that allows users to generate ad hoc networks by adjusting selection criteria (e.g., pair prevalence or association risk ratio), threshold values, and sex, available at: [https://ds3-siscat.github.io/multimorbidity-progression/]. The page also enables customization of display parameters, such as the size range of nodes and edges.

### Additional statistical methods

Owing to the population-based approach, with no sampling strategies applied, no formal sample size estimate was conducted. The cohort characteristics at baseline and at the end of the study period were summarized using counts and percentages for categorical variables and mean and standard deviation (SD) or median and interquartile range (IQR, defined by the 25th and 75th percentiles) for quantitative variables. Besides the most frequent comorbidities, we reported the count and percentage of the most frequent pairs and triads or single conditions. Percentages were estimated over the entire cohort, except otherwise stated.

Chronic conditions were identified using the Chronic Condition Indicator (CCI) developed by the Agency for Healthcare Research and Quality (AHRQ)^42^, which classifies each ICD-10-CM diagnosis code as chronic or acute according to its expected duration and impact on the patient’s health status. Conditions were further coded according to the Clinical Classification Software (CCS) categories^43^. The CCS aggregates all ICD-10-CM diagnosis codes into clinically meaningful groups, facilitating interpretability and reducing dimensionality. In this analysis, we used the 2019.1 version of CCS for ICD-10-CM, which comprises 497 diagnostic groups across 22 body systems.

All analyses were conducted using R statistical package (version 4.4.2)^44^. The visNetwork package^42^ was used to create dynamic HTML widgets for network visualization as Supplementary material.

## Supplementary information

Supplementary information

## Data Availability

The datasets generated and/or analysed during the current study are not publicly available due to their sensitivity as health data, which were de-identified for analysis but not anonymized using irreversible techniques that would preclude any potential re-identification. However, the data can be accessed upon request to the Data Analytics Program for Health Research and Innovation of the Healthcare Quality and Evaluation Agency of Catalonia (AQuAS).

## Code availability

Source codes of the predictive models reported in this article were generated with R Studio (version 4.4.2) and are publicly available at: https://github.com/DS3-SISCAT/multimorbidity-progression.
