# Supplementary material for: Ten-year population-based assessment of multimorbidity burden progression in a regional cohort of 5.5 million adults
Source: NPJ Digit Med. 2026 Jan 31;9:200. doi: 10.1038/s41746-026-02395-x (PMC12963447; doi:10.1038/s41746-026-02395-x)
Supplement: Supplementary file 1 — Supplementary information [file 41746_2026_2395_MOESM1_ESM.pdf]

Ten-year population-based assessment of multimorbidity burden progression in a regional cohort of 5.5 million adults

Supplementary Information

Contents

Supplementary Tables 2

Table S1. Prevalence of ICD-10-CM codes. 2

Table S2. Predictive performance of the models 9

Table S3. Variable importance of the models predicting transition from low/moderate health risk to high/very high risk 10

Table S4. Frequency of the most frequent diagnosis pairs at the end of the study period. 11

Table S5. Frequency of the most frequent diagnosis triads at the end of the study period 12

Supplementary figures 13

Figure S1. Sankey diagram for annual transitions between health-risk strata among patients who died during follow-up 13

Figure S2. Sankey diagram for transitions across AMG risk groups 14

Figure S3. Conditions triggering the transition towards high/very-high risk. 15

Figure S4. Performance of the models for predicting transition to high/very-high clinical risk (Adjusted Morbidity Groups [AMG] index $\geq P_{80}$) 16

Figure S5. Distribution of diagnostic pairs (A) and triads (B) 17

Figure S6. General population network by sex 18

Figure S7. Disease-centered networks by sex 20

Figure S8. Overview of study design 24

Figure S9. Processing algorithm of the Adjusted Morbidity Groups 25

TRIPOD Checklist for Prediction Model Development 26

Supplementary Tables

Table S1. Prevalence of ICD-10-CM codes.

Counts and percentages correspond to ICD-10-CM codes at the end of the investigation period.
Only diagnoses with a prevalence ≥1% are shown.

CCS Code and description	ICD-10-CM Code and description	No. (%)
48 - Thyroid disorders	E02 - Subclinical iodine-deficiency hypothyroidism	249,593 (29.2%)
48 - Thyroid disorders	E039 - Hypothyroidism, unspecified	230,256 (27%)
48 - Thyroid disorders	E0590 - Thyrotoxicosis, unspecified without thyrotoxic crisis or storm	81,512 (9.5%)
48 - Thyroid disorders	E041 - Nontoxic single thyroid nodule	78,026 (9.1%)
48 - Thyroid disorders	E042 - Nontoxic multinodular goiter	36,606 (4.3%)
48 - Thyroid disorders	E049 - Nontoxic goiter, unspecified	36,409 (4.3%)
48 - Thyroid disorders	E069 - Thyroiditis, unspecified	28,623 (3.4%)
48 - Thyroid disorders	E032 - Hypothyroidism due to medicaments and other exogenous substances	21,282 (2.5%)
48 - Thyroid disorders	E031 - Congenital hypothyroidism without goiter	20,404 (2.4%)
48 - Thyroid disorders	E063 - Autoimmune thyroiditis	17,770 (2.1%)
48 - Thyroid disorders	E038 - Other specified hypothyroidism	16,449 (1.9%)
49 - Diabetes mellitus without complication	E119 - Type 2 diabetes mellitus without complications	594,132 (92.5%)
49 - Diabetes mellitus without complication	E109 - Type 1 diabetes mellitus without complications	26,182 (4.1%)
49 - Diabetes mellitus without complication	E139 - Other specified diabetes mellitus without complications	21,992 (3.4%)
53 - Disorders of lipid metabolism	E7800 - Pure hypercholesterolemia, unspecified	652,134 (68.5%)
53 - Disorders of lipid metabolism	E781 - Pure hyperglyceridemia	188,868 (19.8%)
53 - Disorders of lipid metabolism	E782 - Mixed hyperlipidemia	65,355 (6.9%)
53 - Disorders of lipid metabolism	E785 - Hyperlipidemia, unspecified	29,946 (3.1%)
53 - Disorders of lipid metabolism	E7801 - Familial hypercholesterolemia	11,879 (1.2%)
58 - Other nutritional; endocrine; and metabolic disorders	E669 - Obesity, unspecified	894,053 (50.4%)
58 - Other nutritional; endocrine; and metabolic disorders	E789 - Disorder of lipoprotein metabolism, unspecified	662,356 (37.3%)
58 - Other nutritional; endocrine; and metabolic disorders	E668 - Other obesity	60,485 (3.4%)
58 - Other nutritional; endocrine; and metabolic disorders	E739 - Lactose intolerance, unspecified	48,811 (2.8%)
58 - Other nutritional; endocrine; and metabolic disorders	E804 - Gilbert syndrome	24,503 (1.4%)
84 - Headache; including migraine	G43909 - Migraine, unspecified, not intractable, without status migrainosus	256,559 (47%)
84 - Headache; including migraine	G44209 - Tension-type headache, unspecified, not intractable	214,510 (39.3%)
84 - Headache; including migraine	G43109 - Migraine with aura, not intractable, without status migrainosus	42,237 (7.7%)
84 - Headache; including migraine	G43009 - Migraine without aura, not intractable, without status migrainosus	13,012 (2.4%)
86 - Cataract	H259 - Unspecified age-related cataract	514,743 (91.8%)
86 - Cataract	H269 - Unspecified cataract	33,382 (6%)

88 - Glaucoma	H409 - Unspecified glaucoma	221,569 (82.3%)
88 - Glaucoma	H40009 - Preglaucoma, unspecified, unspecified eye	23,930 (8.9%)
88 - Glaucoma	H40059 - Ocular hypertension, unspecified eye	20,777 (7.7%)
89 - Blindness and vision defects	H547 - Unspecified visual loss	502,382 (94.4%)
89 - Blindness and vision defects	H543 - Unqualified visual loss, both eyes	10,275 (1.9%)
89 - Blindness and vision defects	H5440 - Blindness, one eye, unspecified eye	7,015 (1.3%)
94 - Other ear and sense organ disorders	H6090 - Unspecified otitis externa, unspecified ear	776,700 (56.4%)
94 - Other ear and sense organ disorders	H9190 - Unspecified hearing loss, unspecified ear	453,675 (32.9%)
94 - Other ear and sense organ disorders	H905 - Unspecified sensorineural hearing loss	45,074 (3.3%)
94 - Other ear and sense organ disorders	H6240 - Otitis externa in other diseases classified elsewhere, unspecified ear	31,267 (2.3%)
94 - Other ear and sense organ disorders	H902 - Conductive hearing loss, unspecified	25,926 (1.9%)
94 - Other ear and sense organ disorders	H908 - Mixed conductive and sensorineural hearing loss, unspecified	19,649 (1.4%)
94 - Other ear and sense organ disorders	H608X9 - Other otitis externa, unspecified ear	15,728 (1.1%)
95 - Other nervous system disorders	G5600 - Carpal tunnel syndrome, unspecified upper limb	304,163 (51.7%)
95 - Other nervous system disorders	G8929 - Other chronic pain	51,150 (8.7%)
95 - Other nervous system disorders	R262 - Difficulty in walking, not elsewhere classified	41,095 (7%)
95 - Other nervous system disorders	G629 - Polyneuropathy, unspecified	34,860 (5.9%)
95 - Other nervous system disorders	G5760 - Lesion of plantar nerve, unspecified lower limb	22,562 (3.8%)
95 - Other nervous system disorders	G219 - Secondary parkinsonism, unspecified	13,722 (2.3%)
95 - Other nervous system disorders	G5710 - Meralgia paresthetica, unspecified lower limb	13,380 (2.3%)
95 - Other nervous system disorders	G919 - Hydrocephalus, unspecified	12,092 (2.1%)
95 - Other nervous system disorders	G64 - Other disorders of peripheral nervous system	8,780 (1.5%)
95 - Other nervous system disorders	G5601 - Carpal tunnel syndrome, right upper limb	7,732 (1.3%)
95 - Other nervous system disorders	G729 - Myopathy, unspecified	7,282 (1.2%)
98 - Essential hypertension	I10 - Essential (primary) hypertension	1,494,457 (100%)
106 - Cardiac dysrhythmias	I4891 - Unspecified atrial fibrillation	292,126 (52.9%)
106 - Cardiac dysrhythmias	I499 - Cardiac arrhythmia, unspecified	103,964 (18.8%)
106 - Cardiac dysrhythmias	I4949 - Other premature depolarization	32,073 (5.8%)
106 - Cardiac dysrhythmias	I4940 - Unspecified premature depolarization	29,699 (5.4%)
106 - Cardiac dysrhythmias	I493 - Ventricular premature depolarization	28,749 (5.2%)
106 - Cardiac dysrhythmias	I498 - Other specified cardiac arrhythmias	18,530 (3.4%)
106 - Cardiac dysrhythmias	I480 - Paroxysmal atrial fibrillation	15,958 (2.9%)
106 - Cardiac dysrhythmias	I4892 - Unspecified atrial flutter	13,046 (2.4%)
106 - Cardiac dysrhythmias	I479 - Paroxysmal tachycardia, unspecified	11,360 (2.1%)

127 - Chronic obstructive pulmonary disease and bronchiectasis	J449 - Chronic obstructive pulmonary disease, unspecified	219,601 (54.1%)
127 - Chronic obstructive pulmonary disease and bronchiectasis	J42 - Unspecified chronic bronchitis	63,004 (15.5%)
127 - Chronic obstructive pulmonary disease and bronchiectasis	J479 - Bronchiectasis, uncomplicated	55,697 (13.7%)
127 - Chronic obstructive pulmonary disease and bronchiectasis	J439 - Emphysema, unspecified	25,677 (6.3%)
127 - Chronic obstructive pulmonary disease and bronchiectasis	J441 - Chronic obstructive pulmonary disease with (acute) exacerbation	25,523 (6.3%)
127 - Chronic obstructive pulmonary disease and bronchiectasis	J410 - Simple chronic bronchitis	6,346 (1.6%)
127 - Chronic obstructive pulmonary disease and bronchiectasis	J418 - Mixed simple and mucopurulent chronic bronchitis	4,911 (1.2%)
128 - Asthma	J45909 - Unspecified asthma, uncomplicated	410,801 (97.3%)
128 - Asthma	J45902 - Unspecified asthma with status asthmaticus	8,500 (2%)
134 - Other upper respiratory disease	J309 - Allergic rhinitis, unspecified	705,773 (76.6%)
134 - Other upper respiratory disease	J300 - Vasomotor rhinitis	68,169 (7.4%)
134 - Other upper respiratory disease	J3089 - Other allergic rhinitis	54,259 (5.9%)
134 - Other upper respiratory disease	J312 - Chronic pharyngitis	53,405 (5.8%)
134 - Other upper respiratory disease	J310 - Chronic rhinitis	17,274 (1.9%)
134 - Other upper respiratory disease	J301 - Allergic rhinitis due to pollen	10,706 (1.2%)
138 - Esophageal disorders	K219 - Gastro-esophageal reflux disease without esophagitis	345,445 (90.8%)
138 - Esophageal disorders	K2270 - Barrett's esophagus without dysplasia	14,180 (3.7%)
138 - Esophageal disorders	I8500 - Esophageal varices without bleeding	10,982 (2.9%)
138 - Esophageal disorders	K224 - Dyskinesia of esophagus	5,802 (1.5%)
151 - Other liver diseases	K760 - Fatty (change of) liver, not elsewhere classified	210,227 (64.5%)
151 - Other liver diseases	K7689 - Other specified diseases of liver	32,374 (9.9%)
151 - Other liver diseases	K769 - Liver disease, unspecified	31,185 (9.6%)
151 - Other liver diseases	K7460 - Unspecified cirrhosis of liver	22,397 (6.9%)
151 - Other liver diseases	K759 - Inflammatory liver disease, unspecified	10,215 (3.1%)
151 - Other liver diseases	K7589 - Other specified inflammatory liver diseases	5,650 (1.7%)
151 - Other liver diseases	K766 - Portal hypertension	5,614 (1.7%)
163 - Genitourinary symptoms and ill-defined conditions	R32 - Unspecified urinary incontinence	573,504 (78.5%)
163 - Genitourinary symptoms and ill-defined conditions	N39498 - Other specified urinary incontinence	57,408 (7.9%)

163 - Genitourinary symptoms and ill-defined conditions	N393 - Stress incontinence (female) (male)	53,359 (7.3%)
163 - Genitourinary symptoms and ill-defined conditions	N3941 - Urge incontinence	33,002 (4.5%)
163 - Genitourinary symptoms and ill-defined conditions	N3946 - Mixed incontinence	9,731 (1.3%)
164 - Hyperplasia of prostate	N400 - Benign prostatic hyperplasia without lower urinary tract symptoms	399,488 (99.6%)
171 - Menstrual disorders	N921 - Excessive and frequent menstruation with irregular cycle	187,005 (24.6%)
171 - Menstrual disorders	N912 - Amenorrhea, unspecified	144,856 (19%)
171 - Menstrual disorders	N946 - Dysmenorrhea, unspecified	139,013 (18.3%)
171 - Menstrual disorders	N920 - Excessive and frequent menstruation with regular cycle	121,979 (16%)
171 - Menstrual disorders	N926 - Irregular menstruation, unspecified	67,498 (8.9%)
171 - Menstrual disorders	N911 - Secondary amenorrhea	66,380 (8.7%)
171 - Menstrual disorders	N944 - Primary dysmenorrhea	14,155 (1.9%)
173 - Menopausal disorders	N951 - Menopausal and female climacteric states	142,428 (42%)
173 - Menopausal disorders	N950 - Postmenopausal bleeding	70,225 (20.7%)
173 - Menopausal disorders	N959 - Unspecified menopausal and perimenopausal disorder	55,631 (16.4%)
173 - Menopausal disorders	N952 - Postmenopausal atrophic vaginitis	37,312 (11%)
173 - Menopausal disorders	E28319 - Asymptomatic premature menopause	13,552 (4%)
173 - Menopausal disorders	E2839 - Other primary ovarian failure	12,621 (3.7%)
173 - Menopausal disorders	N924 - Excessive bleeding in the premenopausal period	4,599 (1.4%)
198 - Other inflammatory condition of skin	L409 - Psoriasis, unspecified	147,076 (43.4%)
198 - Other inflammatory condition of skin	L719 - Rosacea, unspecified	59,973 (17.7%)
198 - Other inflammatory condition of skin	L42 - Pityriasis rosea	59,745 (17.6%)
198 - Other inflammatory condition of skin	L305 - Pityriasis alba	22,418 (6.6%)
198 - Other inflammatory condition of skin	L404 - Guttate psoriasis	13,150 (3.9%)
198 - Other inflammatory condition of skin	L4059 - Other psoriatic arthropathy	8,519 (2.5%)
198 - Other inflammatory condition of skin	L4050 - Arthropathic psoriasis, unspecified	8,039 (2.4%)
203 - Osteoarthritis	M179 - Osteoarthritis of knee, unspecified	424,129 (35.4%)
203 - Osteoarthritis	M1990 - Unspecified osteoarthritis, unspecified site	256,086 (21.4%)
203 - Osteoarthritis	M169 - Osteoarthritis of hip, unspecified	171,155 (14.3%)
203 - Osteoarthritis	M159 - Polyosteoarthritis, unspecified	138,090 (11.5%)
203 - Osteoarthritis	M189 - Osteoarthritis of first carpometacarpal joint, unspecified	73,849 (6.2%)
203 - Osteoarthritis	M1810 - Unilateral primary osteoarthritis of first carpometacarpal joint, unspecified hand	49,524 (4.1%)
203 - Osteoarthritis	M19049 - Primary osteoarthritis, unspecified hand	38,103 (3.2%)
205 - Spondylosis; intervertebral disc disorders; other back problems	M519 - Unspecified thoracic, thoracolumbar and lumbosacral intervertebral disc disorder	258,658 (29.8%)

205 - Spondylosis; intervertebral disc disorders; other back problems	M47899 - Other spondylosis, site unspecified	132,403 (15.2%)
205 - Spondylosis; intervertebral disc disorders; other back problems	M5126 - Other intervertebral disc displacement, lumbar region	127,780 (14.7%)
205 - Spondylosis; intervertebral disc disorders; other back problems	M479 - Spondylosis, unspecified	107,117 (12.3%)
205 - Spondylosis; intervertebral disc disorders; other back problems	M47892 - Other spondylosis, cervical region	56,137 (6.5%)
205 - Spondylosis; intervertebral disc disorders; other back problems	M47896 - Other spondylosis, lumbar region	55,198 (6.4%)
205 - Spondylosis; intervertebral disc disorders; other back problems	M5090 - Cervical disc disorder, unspecified, unspecified cervical region	36,630 (4.2%)
205 - Spondylosis; intervertebral disc disorders; other back problems	M4726 - Other spondylosis with radiculopathy, lumbar region	22,995 (2.6%)
205 - Spondylosis; intervertebral disc disorders; other back problems	M461 - Sacroiliitis, not elsewhere classified	16,828 (1.9%)
205 - Spondylosis; intervertebral disc disorders; other back problems	M5020 - Other cervical disc displacement, unspecified cervical region	16,685 (1.9%)
205 - Spondylosis; intervertebral disc disorders; other back problems	M47894 - Other spondylosis, thoracic region	8,676 (1%)
206 - Osteoporosis	M810 - Age-related osteoporosis without current pathological fracture	151,616 (53.5%)
206 - Osteoporosis	M818 - Other osteoporosis without current pathological fracture	131,511 (46.4%)
208 - Acquired foot deformities	M2010 - Hallux valgus (acquired), unspecified foot	174,297 (80.4%)
208 - Acquired foot deformities	M2040 - Other hammer toe(s) (acquired), unspecified foot	38,938 (18%)
208 - Acquired foot deformities	M2030 - Hallux varus (acquired), unspecified foot	2,318 (1.1%)
209 - Other acquired deformities	M419 - Scoliosis, unspecified	239,324 (87.1%)
209 - Other acquired deformities	M4050 - Lordosis, unspecified, site unspecified	9,858 (3.6%)
209 - Other acquired deformities	M4000 - Postural kyphosis, site unspecified	8,627 (3.1%)
209 - Other acquired deformities	M40209 - Unspecified kyphosis, site unspecified	8,250 (3%)
209 - Other acquired deformities	M40204 - Unspecified kyphosis, thoracic region	5,345 (1.9%)
225 - Joint disorders and dislocations; trauma-related	M1250 - Traumatic arthropathy, unspecified site	183,063 (40.6%)
225 - Joint disorders and dislocations; trauma-related	M23309 - Other meniscus derangements, unspecified meniscus, unspecified knee	146,812 (32.5%)
225 - Joint disorders and dislocations; trauma-related	M2240 - Chondromalacia patellae, unspecified knee	61,835 (13.7%)
225 - Joint disorders and dislocations; trauma-related	M2350 - Chronic instability of knee, unspecified knee	25,293 (5.6%)
225 - Joint disorders and dislocations; trauma-related	M2390 - Unspecified internal derangement of unspecified knee	11,129 (2.5%)
225 - Joint disorders and dislocations; trauma-related	M222X9 - Patellofemoral disorders, unspecified knee	5,821 (1.3%)
225 - Joint disorders and dislocations; trauma-related	M238X9 - Other internal derangements of unspecified knee	4,852 (1.1%)
253 - Allergic reactions	L209 - Atopic dermatitis, unspecified	482,466 (96.9%)
253 - Allergic reactions	L2089 - Other atopic dermatitis	7,377 (1.5%)
650 - Adjustment disorders	F4320 - Adjustment disorder, unspecified	254,969 (86.8%)
650 - Adjustment disorders	F439 - Reaction to severe stress, unspecified	16,453 (5.6%)
650 - Adjustment disorders	F4321 - Adjustment disorder with depressed mood	11,407 (3.9%)

650 - Adjustment disorders	F4323 - Adjustment disorder with mixed anxiety and depressed mood	6,187 (2.1%)
650 - Adjustment disorders	F4322 - Adjustment disorder with anxiety	3,283 (1.1%)
651 - Anxiety disorders	F419 - Anxiety disorder, unspecified	1,396,954 (68.9%)
651 - Anxiety disorders	F413 - Other mixed anxiety disorders	285,065 (14.1%)
651 - Anxiety disorders	F411 - Generalized anxiety disorder	141,292 (7%)
651 - Anxiety disorders	F410 - Panic disorder [episodic paroxysmal anxiety]	72,812 (3.6%)
651 - Anxiety disorders	F409 - Phobic anxiety disorder, unspecified	26,755 (1.3%)
651 - Anxiety disorders	F429 - Obsessive-compulsive disorder, unspecified	21,288 (1.1%)
657 - Mood disorders	F329 - Major depressive disorder, single episode, unspecified	547,076 (67.2%)
657 - Mood disorders	F341 - Dysthymic disorder	80,277 (9.9%)
657 - Mood disorders	F322 - Major depressive disorder, single episode, severe without psychotic features	42,479 (5.2%)
657 - Mood disorders	F339 - Major depressive disorder, recurrent, unspecified	32,495 (4%)
657 - Mood disorders	F320 - Major depressive disorder, single episode, mild	21,461 (2.6%)
657 - Mood disorders	F319 - Bipolar disorder, unspecified	20,673 (2.5%)
657 - Mood disorders	F39 - Unspecified mood [affective] disorder	14,239 (1.7%)
657 - Mood disorders	F321 - Major depressive disorder, single episode, moderate	11,007 (1.4%)
657 - Mood disorders	F332 - Major depressive disorder, recurrent severe without psychotic features	9,855 (1.2%)
657 - Mood disorders	F349 - Persistent mood [affective] disorder, unspecified	8,305 (1%)
660 - Alcohol-related disorders	F1010 - Alcohol abuse, uncomplicated	77,639 (36.8%)
660 - Alcohol-related disorders	F1020 - Alcohol dependence, uncomplicated	65,966 (31.2%)
660 - Alcohol-related disorders	F1019 - Alcohol abuse with unspecified alcohol-induced disorder	34,261 (16.2%)
660 - Alcohol-related disorders	K7010 - Alcoholic hepatitis without ascites	8,310 (3.9%)
660 - Alcohol-related disorders	K709 - Alcoholic liver disease, unspecified	6,548 (3.1%)
660 - Alcohol-related disorders	K7030 - Alcoholic cirrhosis of liver without ascites	4,824 (2.3%)
660 - Alcohol-related disorders	G621 - Alcoholic polyneuropathy	3,914 (1.9%)
660 - Alcohol-related disorders	F1021 - Alcohol dependence, in remission	3,371 (1.6%)
660 - Alcohol-related disorders	F10239 - Alcohol dependence with withdrawal, unspecified	2,127 (1%)
661 - Substance-related disorders	F17200 - Nicotine dependence, unspecified, uncomplicated	851,228 (87.7%)
661 - Substance-related disorders	F1420 - Cocaine dependence, uncomplicated	26,945 (2.8%)
661 - Substance-related disorders	F1219 - Cannabis abuse with unspecified cannabis-induced disorder	15,124 (1.6%)
661 - Substance-related disorders	F1220 - Cannabis dependence, uncomplicated	14,984 (1.5%)
661 - Substance-related disorders	F1920 - Other psychoactive substance dependence, uncomplicated	11,385 (1.2%)
670 - Miscellaneous mental health disorders	F5109 - Other insomnia not due to a substance or known physiological condition	609,111 (65.2%)
670 - Miscellaneous mental health disorders	F5221 - Male erectile disorder	121,821 (13%)
670 - Miscellaneous mental health disorders	F458 - Other somatoform disorders	38,588 (4.1%)

670 - Miscellaneous mental health disorders	F509 - Eating disorder, unspecified	24,398 (2.6%)
670 - Miscellaneous mental health disorders	F529 - Unspecified sexual dysfunction not due to a substance or known physiological condition	20,822 (2.2%)
670 - Miscellaneous mental health disorders	F524 - Premature ejaculation	13,269 (1.4%)
670 - Miscellaneous mental health disorders	F502 - Bulimia nervosa	11,561 (1.2%)
670 - Miscellaneous mental health disorders	F4520 - Hypochondriacal disorder, unspecified	10,917 (1.2%)

CCS: Clinical Classification Software for chronic diseases. **ICD-10-CM**: International Classification of Diseases, version 10, Clinical Modification.

Table S2. Predictive performance of the models

Base models, including only age and sex; Complexity models**,** additionally incorporating AMG-based multimorbidity indicators and timing of diagnoses; and Full-disease models, which further include binary indicators for the presence of each chronic condition. Each specification was tested using logistic regression, random forest, neural networks, and extreme gradient boosting.

Model type	Algorithm	AUC-ROC	AUC-PR	Sensitivity	Specificity	Accuracy
Base	Logistic Regression	0.8480	0.5964	0.5785	0.9415	0.6512
Base	Random Forest	0.8138	0.4459	0.7399	0.8604	0.7640
Base	Neural Networks	0.8799	0.6400	0.7782	0.8260	0.7878
Base	Extreme Gradient Boosting	0.8800	0.6432	0.7782	0.8260	0.7878
Complexity	Logistic Regression	0.9387	0.8372	0.8651	0.8558	0.8632
Complexity	Random Forest	0.9282	0.8213	0.8471	0.8685	0.8514
Complexity	Neural Networks	0.9388	0.8378	0.8588	0.8626	0.8595
Complexity	Extreme Gradient Boosting	0.9395	0.8391	0.859	0.8644	0.8601
Full-disease	Logistic Regression	0.9365	0.8335	0.8759	0.8387	0.8684
Full-disease	Random Forest	0.9292	0.8191	0.8439	0.8716	0.8494
Full-disease	Neural Networks	0.9294	0.7945	0.8952	0.7923	0.8746
Full-disease	Extreme Gradient Boosting	0.9397	0.8396	0.8582	0.8661	0.8597

Table S3. Variable importance of the models predicting transition from low/moderate health risk to high/very high risk

Variable importance was computed separately for each model (GLM, RF, NNET, XGBoost) and normalized to a 0–1 scale within each model. For each model, the top 35 most influential features were selected. As no meaningful differences were found between the complex and full-disease models, we used the full-disease model to maximize the number of variables and to compare the multimorbidity index with individual chronic-disease indicators. Color intensity represents the gradient between 0 (minimal contribution to transition) and 1 (highest contribution to transition).

Feature	GLM	NNET	RF	XGB
Multimorbidity burden (AMG index)	1,000	0,806	0,876	1,000
Age	0,149	0,580	1,000	0,287
Number of chronic conditions	0,477	1,000	0,361	0,001
AMG index at previous disease	0,393	0,285	0,588	0,001
Sex	0,178	0,207	0,399	0,002
AMG index at first disease	0,255	0,134	0,348	0,001
Time since onset of previous disease (years)	0,008	0,146	0,427	0,002
Substance-related disorders	0,007	-	0,235	0,001
Time since onset of first disease (years)	0,015	-	0,222	0,001
Essential hypertension	0,004	-	0,216	0,001
Hypertension with complications and secondary hypertension	-	0,194	-	-
Diabetes mellitus without complication	0,007	-	0,180	0,001
Anxiety disorders	-	-	0,187	-
Other nutritio-l; endocrine; and metabolic disorders	0,006	-	0,167	0,000
Cardiac dysrhythmias	-	0,157	-	-
Osteoporosis	0,015	0,000	0,122	0,000
Osteoarthritis	0,008	-	0,124	-
Thyroid disorders	0,006	-	0,125	-
Spondylosis; intervertebral disc disorders; other back problems	-	-	0,128	-
Asthma	-	-	0,127	-
Miscellaneous mental health disorders	-	-	0,126	-
Disorders of lipid metabolism	0,004	-	0,119	-
Pulmo-ry heart disease	-	0,121	-	-
Chronic kidney disease	-	0,103	-	-
Cancer of bronchus; lung	-	0,075	-	-
Secondary malig-ncies	-	0,065	-	-
Chronic obstructive pulmo-ry disease and bronchiectasis	-	0,044	-	-
Cancer of colon	-	0,029	-	-
Hyperplasia of prostate	0,013	-	-	0,000
Cataract	0,012	-	-	-
Coro-ry atherosclerosis and other heart disease	-	0,012	-	-
Other upper respiratory disease	0,010	-	-	0,000
Acute cerebrovascular disease	-	0,006	-	-
Other ear and sense organ disorders	0,006	-	-	-
Acute myocardial infarction	-	0,004	-	-

Table S4. Frequency of the most frequent diagnosis pairs at the end of the study period.

Disease 1	Disease 2	Frequency	Prevalence	Risk Ratio
Other nutritional; endocrine; and metabolic disorders	Essential hypertension	686,161	12.4%	2.08
Other nutritional; endocrine; and metabolic disorders	Anxiety disorders	430,892	7.8%	1.27
Essential hypertension	Osteoarthritis	392,135	7.1%	2.44
Essential hypertension	Anxiety disorders	369,310	6.7%	1.17
Other nutritional; endocrine; and metabolic disorders	Osteoarthritis	357,420	6.5%	2.07
Diabetes mellitus without complication	Essential hypertension	344,076	6.2%	2.85
Anxiety disorders	Substance-related disorders	330,108	6.0%	1.46
Diabetes mellitus without complication	Other nutritional; endocrine; and metabolic disorders	309,825	5.6%	2.38
Other nutritional; endocrine; and metabolic disorders	Substance-related disorders	286,014	5.2%	1.21
Anxiety disorders	Mood disorders	280,705	5.1%	2.00
Anxiety disorders	Miscellaneous mental health disorders	246,878	4.5%	1.78
Thyroid disorders	Other nutritional; endocrine; and metabolic disorders	238,095	4.3%	1.60
Essential hypertension	Substance-related disorders	238,041	4.3%	1.08
Other nutritional; endocrine; and metabolic disorders	Mood disorders	237,057	4.3%	1.62
Essential hypertension	Miscellaneous mental health disorders	235,151	4.2%	1.74

Table S5. Frequency of the most frequent diagnosis triads at the end of the study period

Disease 1	Disease 2	Disease 3	Frequency	Prevalence	Risk Ratio*
Other nutritional; endocrine; and metabolic disorders	Essential hypertension	Osteoarthritis	240,643	4.3%	5.91
Diabetes mellitus without complication	Other nutritional; endocrine; and metabolic disorders	Essential hypertension	225,953	4.1%	7.39
Other nutritional; endocrine; and metabolic disorders	Essential hypertension	Anxiety disorders	204,517	3.7%	2.55
Other nutritional; endocrine; and metabolic disorders	Essential hypertension	Spondylosis; intervertebral disc disorders; other back problems	134,277	2.4%	4.14
Other nutritional; endocrine; and metabolic disorders	Essential hypertension	Miscellaneous mental health disorders	133,760	2.4%	3.92
Other nutritional; endocrine; and metabolic disorders	Essential hypertension	Mood disorders	130,592	2.4%	3.78
Other nutritional; endocrine; and metabolic disorders	Cataract	Essential hypertension	129,728	2.3%	6.02
Other nutritional; endocrine; and metabolic disorders	Essential hypertension	Substance-related disorders	129,102	2.3%	2.32
Essential hypertension	Osteoarthritis	Anxiety disorders	127,455	2.3%	3.27
Thyroid disorders	Other nutritional; endocrine; and metabolic disorders	Essential hypertension	122,957	2.2%	3.50
Other nutritional; endocrine; and metabolic disorders	Osteoarthritis	Anxiety disorders	121,482	2.2%	2.89
Diabetes mellitus without complication	Essential hypertension	Osteoarthritis	117,456	2.1%	7.88
Other nutritional; endocrine; and metabolic disorders	Essential hypertension	Genitourinary symptoms and ill-defined conditions	114,850	2.1%	5.79
Other nutritional; endocrine; and metabolic disorders	Anxiety disorders	Mood disorders	113,210	2.0%	3.18
Cataract	Essential hypertension	Osteoarthritis	112,393	2.0%	10.69

* The risk ratio (RR) for the combination of three conditions was estimated as follows: RR = [No. of individuals with the triad] × [total population]^2 / (N1×N2×N3) , where N1, N2, and N3 correspond to the no. of individuals with each of the conditions within the triad.

Supplementary figures

Figure S1. Sankey diagram for annual transitions between health-risk strata among patients who died during follow-up

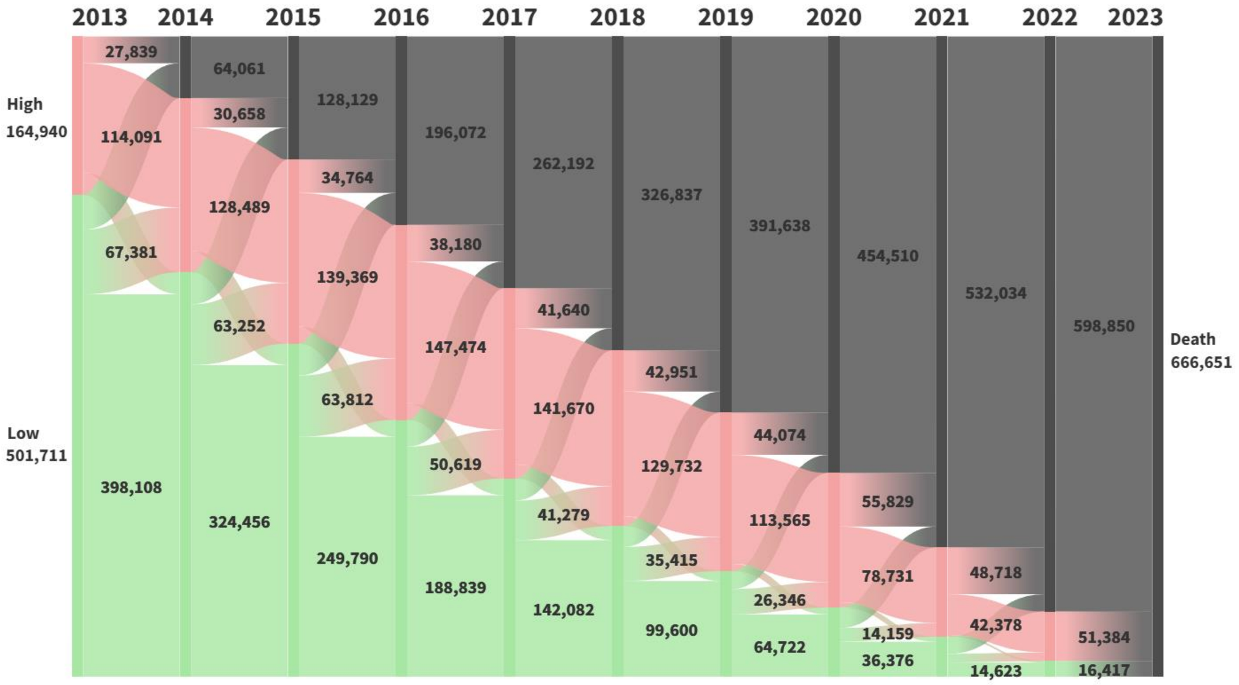

Trajectories preceding death among the subset of individuals who died within the analysis period (i.e., from January 01, 2013 to December 31, 2023); percentages are estimated over the entire cohort (N= 6,205,308):

- Low/moderate risk → death: 210,800 (3.40%)
- Low/moderate risk → high/very high risk → death: 319,834 (5.15%)
- High/very high risk → death: 136,017 (2.19%)

Figure S2. Sankey diagram for transitions across AMG risk groups

Flow of individuals across Adjusted Morbidity Groups (AMG) risk strata (Low <P50, Moderate P50–80, High P80–95, Very high ≥P95) from baseline to the end of the study period, showing the number of individuals in each group and their transitions over time.

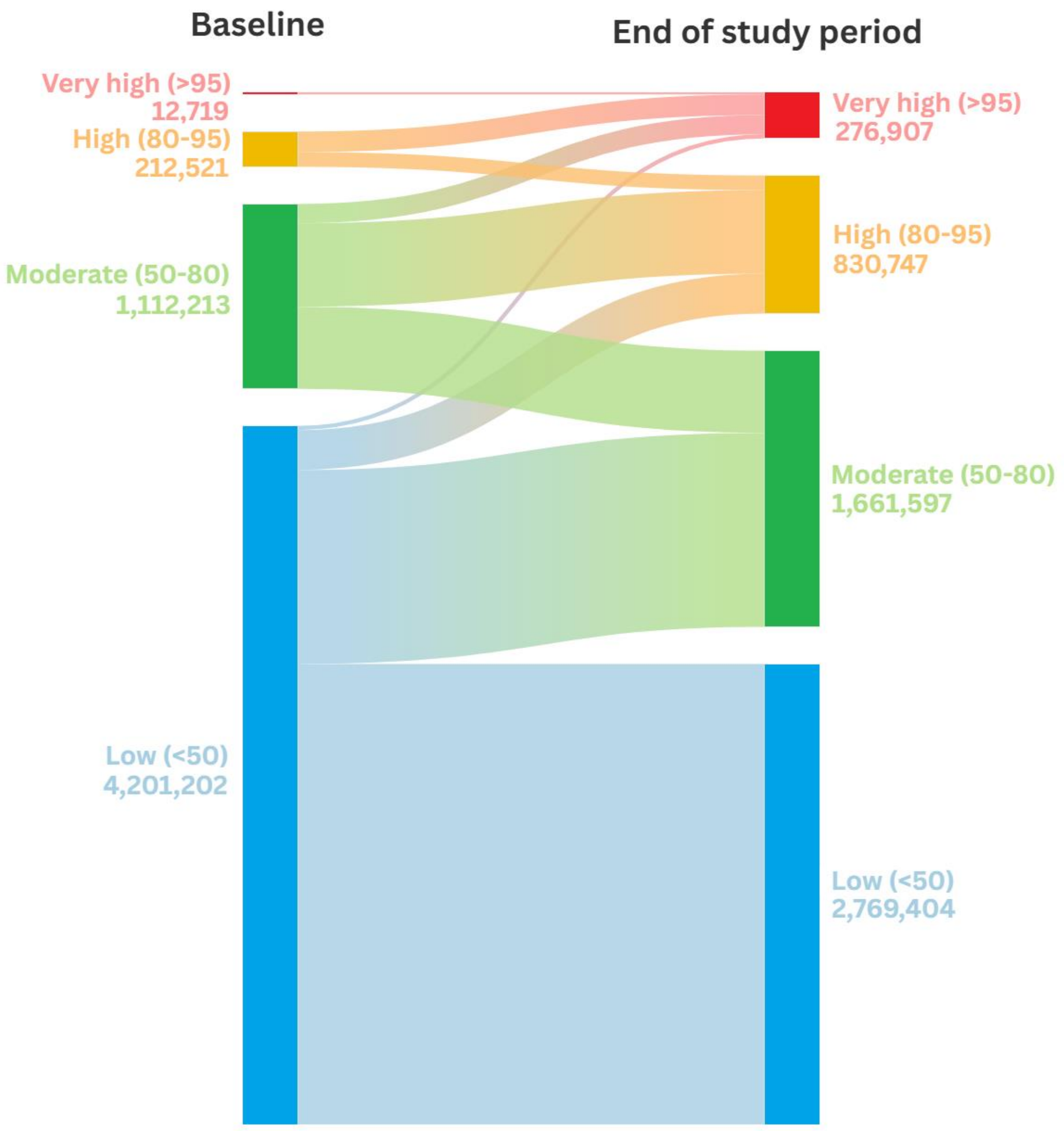

Figure S3. Conditions triggering the transition towards high/very-high risk.

Chronic kidney disease was the most frequent new diagnosis at the time of transition to a higher-risk group (n=59,628; 5.37% of those who progressed), followed by Essential hypertension (n=47,215; 4.25%) and Osteoarthritis (n=41,751; 3.76%).

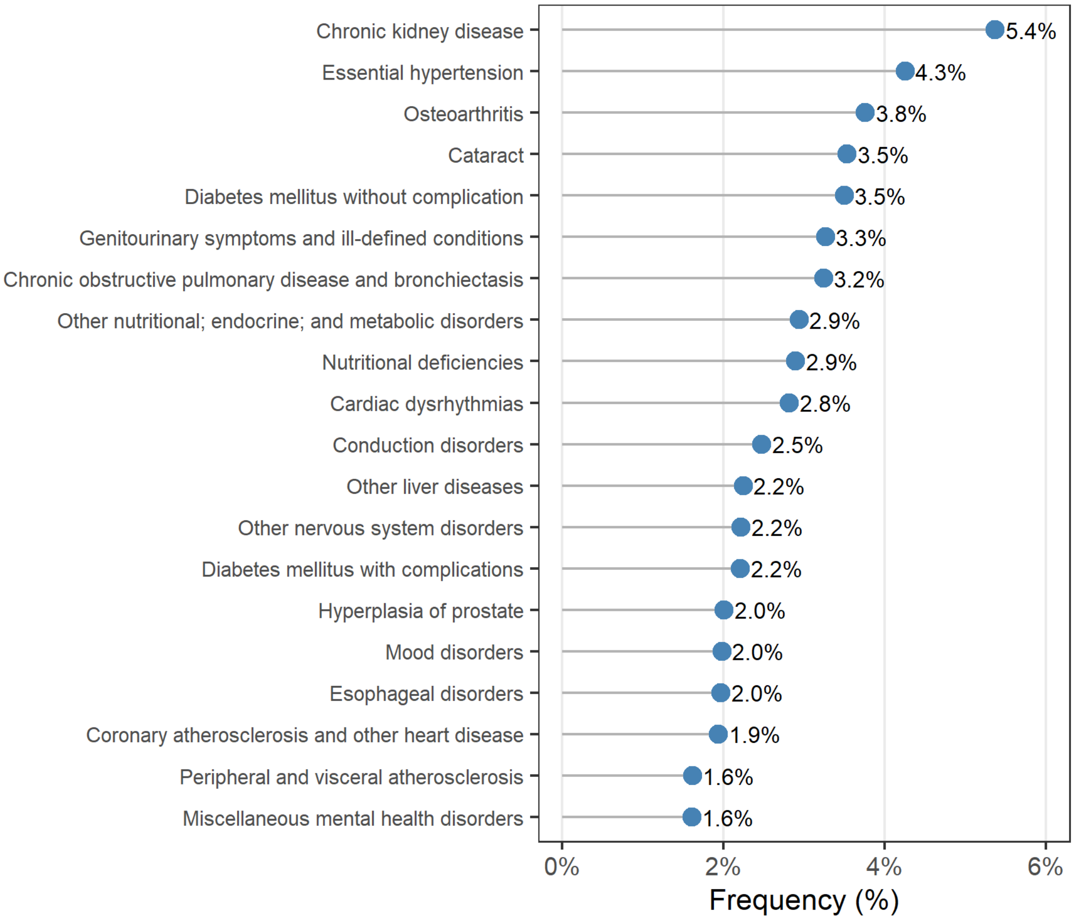

Figure S4. Performance of the models for predicting transition to high/very-high clinical risk (Adjusted Morbidity Groups [AMG] index ≥P_{80})

Point estimates of the area under the precision-recall and ROC curves for the complexity model in the general (solid line) and dedicated (dotted line) forms for the populations used to produce the dedicated models (i.e., individuals with a given disease).

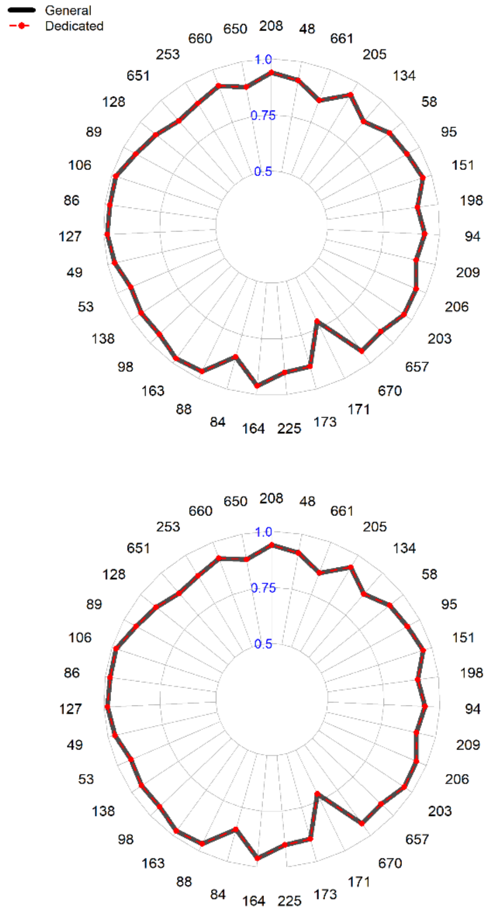

48 — Thyroid disorders

49 — Diabetes mellitus without complication

53 — Disorders of lipid metabolism

58 — Other nutritional; endocrine; and metabolic disorders

84 — Headache; including migraine

86 — Cataract

88 — Glaucoma

89 — Blindness and vision defects

94 — Other ear and sense organ disorders

95 — Other nervous system disorders

98 — Essential hypertension

106 — Cardiac dysrhythmias

127 — Chronic obstructive pulmonary disease and bronchiectasis

128 — Asthma

134 — Other upper respiratory disease

138 — Esophageal disorders

151 — Other liver diseases

163 — Genitourinary symptoms and ill-defined conditions

164 — Hyperplasia of prostate

171 — Menstrual disorders

173 — Menopausal disorders

198 — Other inflammatory condition of skin

203 — Osteoarthritis

205 — Spondylosis; intervertebral disc disorders; other back problems

206 — Osteoporosis

208 — Acquired foot deformities

o

209 — Other acquired deformities

225 — Joint disorders and dislocations; trauma-related

253 — Allergic reactions

650 — Adjustment disorders

651 — Anxiety disorders

657 — Mood disorders

660 — Alcohol-related disorders

661 — Substance-related disorders

670 — Miscellaneous mental health disorders

Figure S5. Distribution of diagnostic pairs (A) and triads (B)

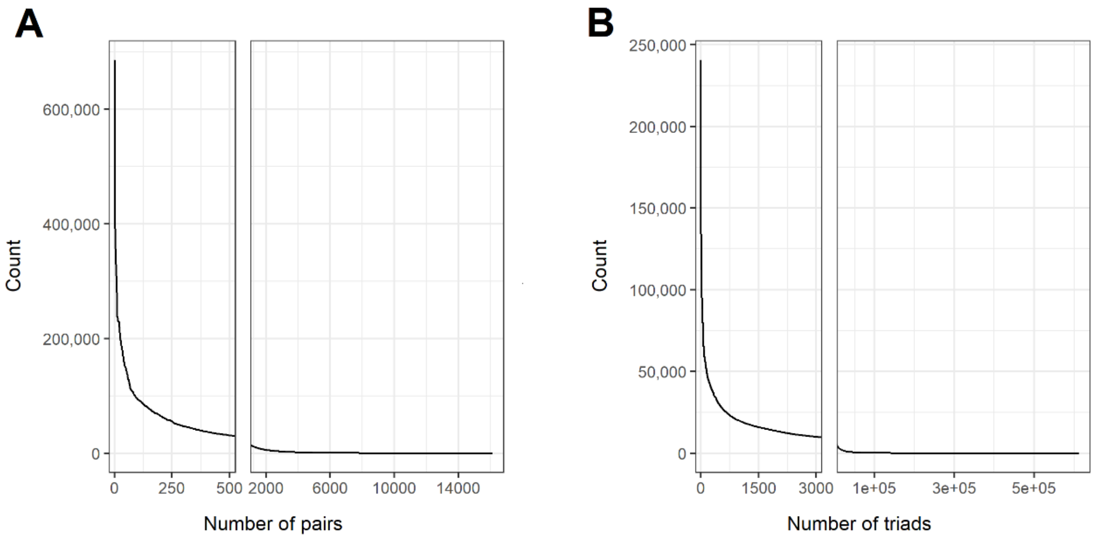

Figure S6. General population network by sex

Dynamic HTML networks are available at: https://ds3-siscat.github.io/multimorbidity-progression/

General population: Female

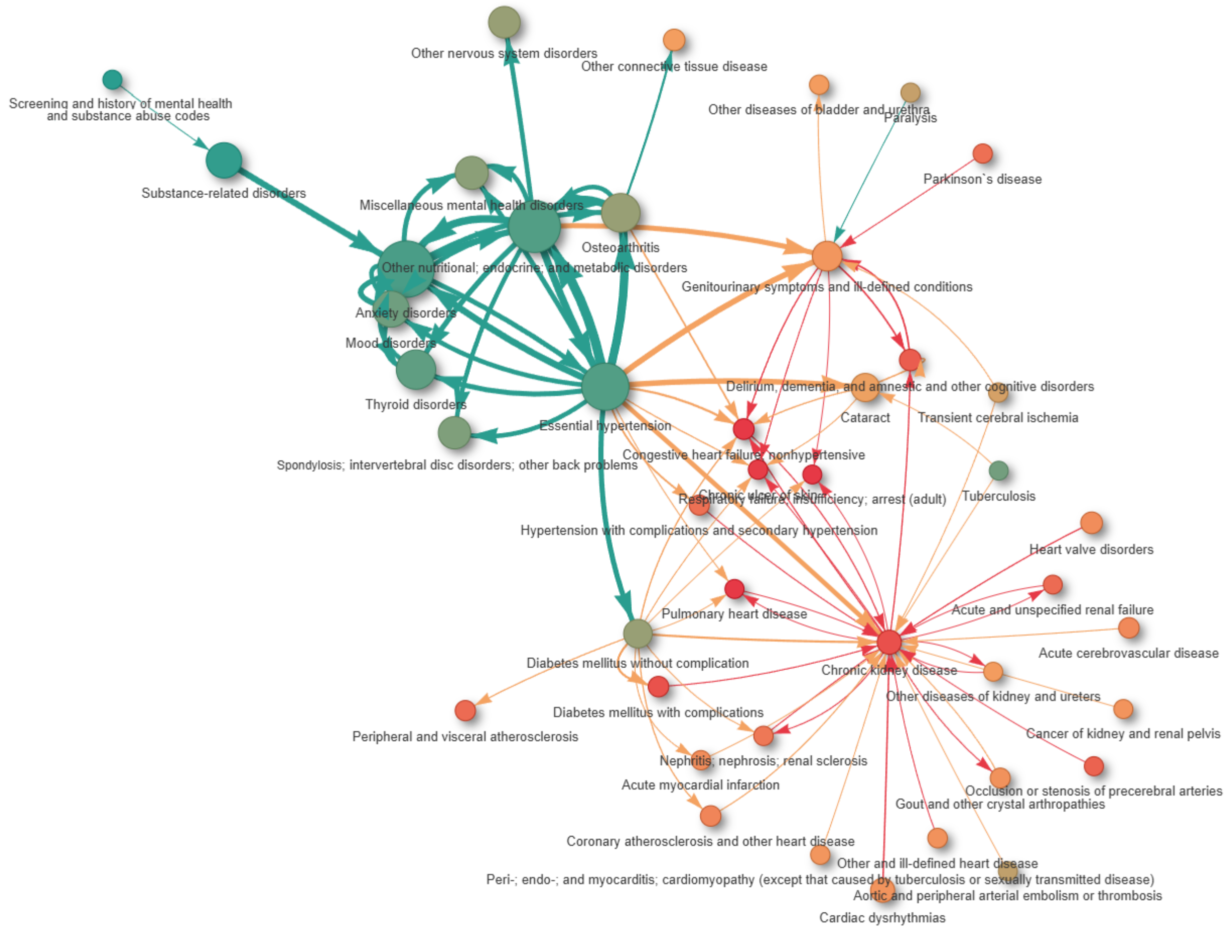

General population: Male

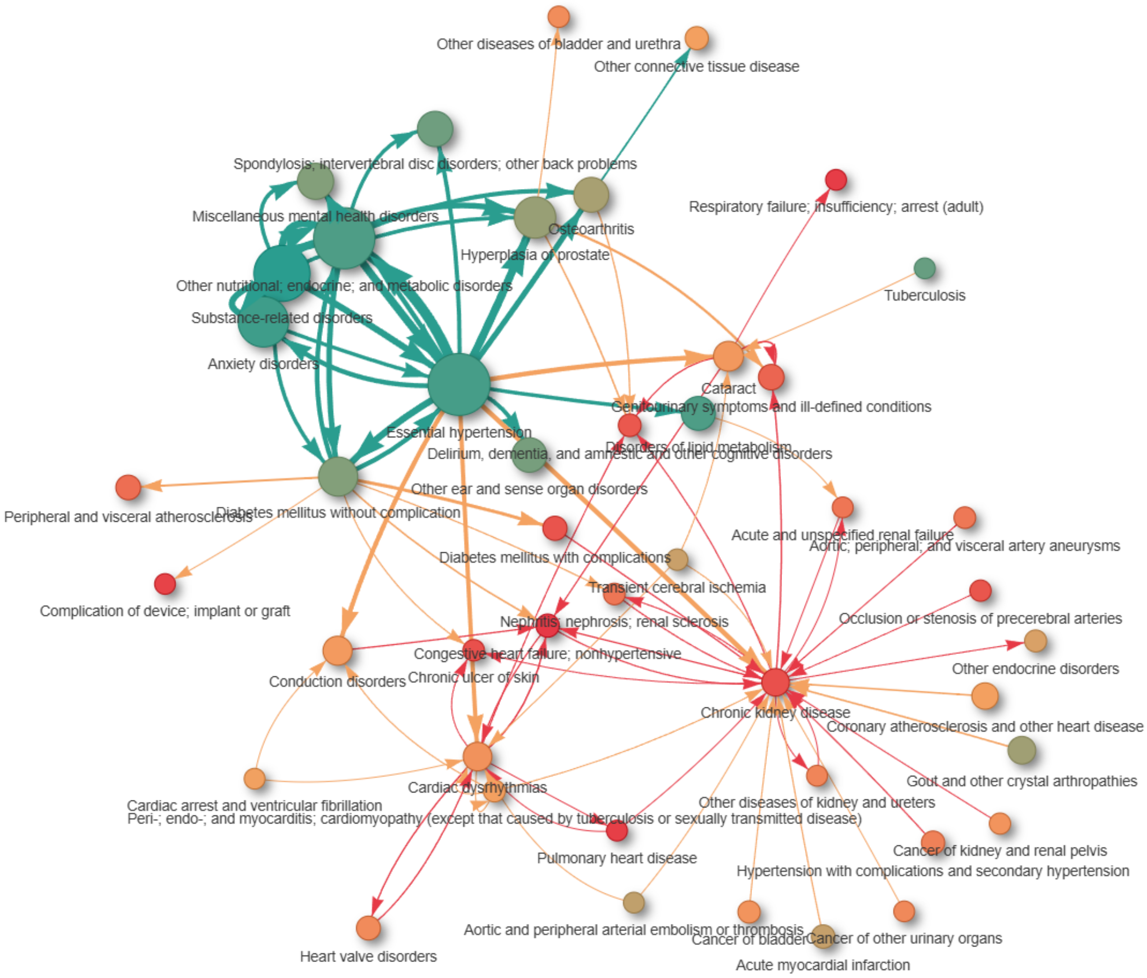

Figure S7. Disease-centered networks by sex

The four disease-centered networks by sex shown in the main paper are displayed here. The remaining 16 disease-centered networks can be explored interactively here:

https://ds3-siscat.github.io/multimorbidity-progression/

Other nutritional; endocrine; and metabolic disorders: Female

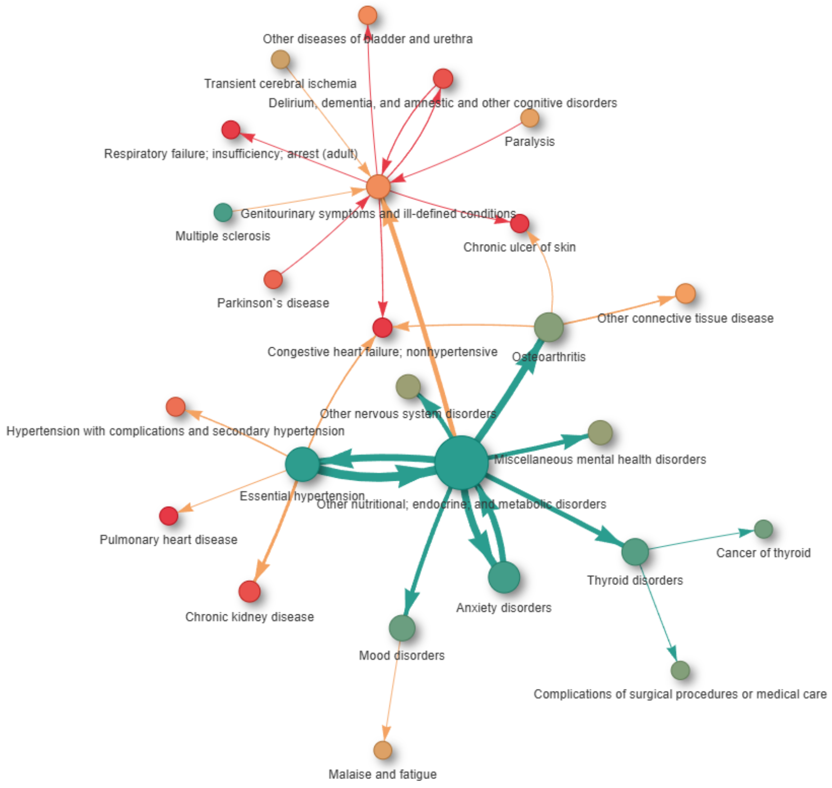

Other nutritional; endocrine; and metabolic disorders: Male

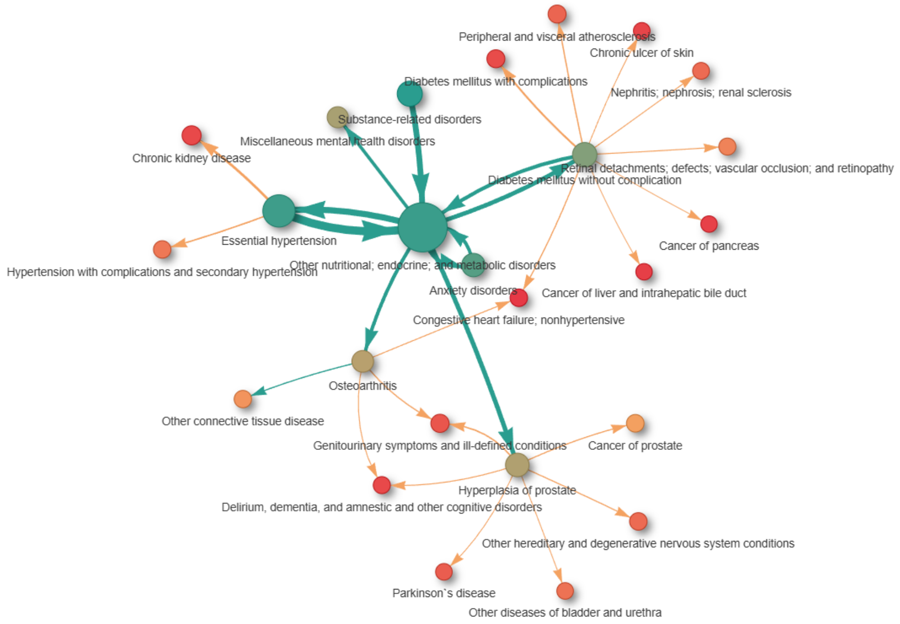

Anxiety Disorders: Female

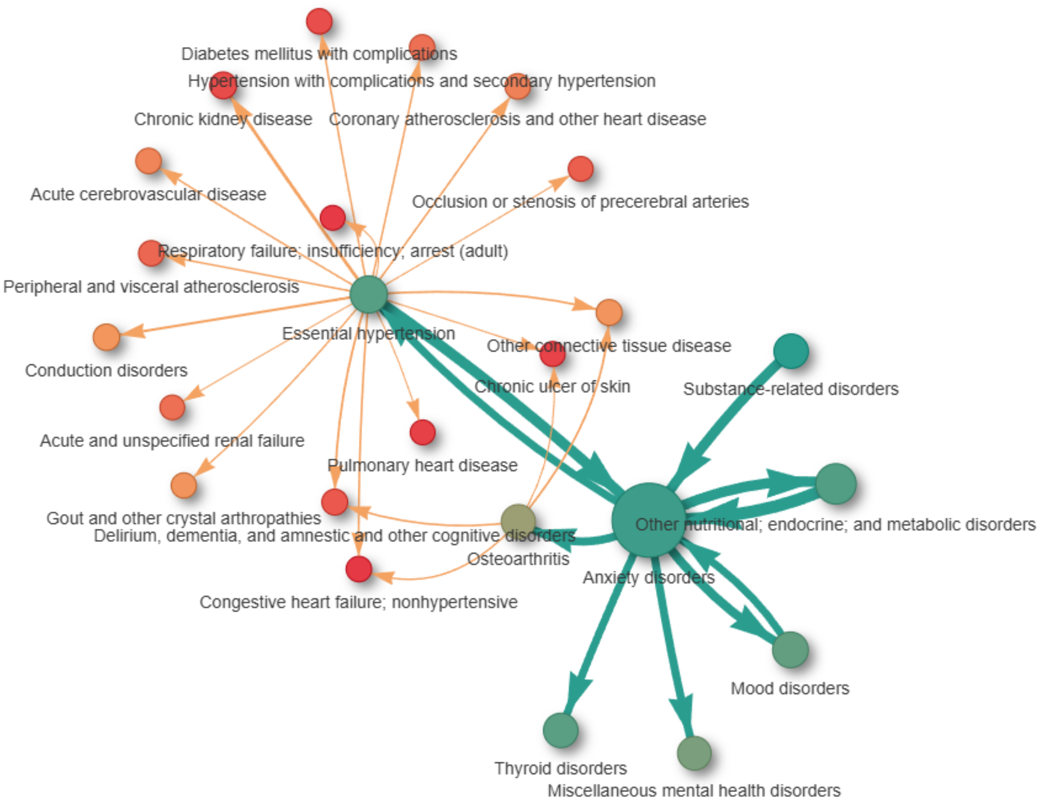

Anxiety Disorders: Male

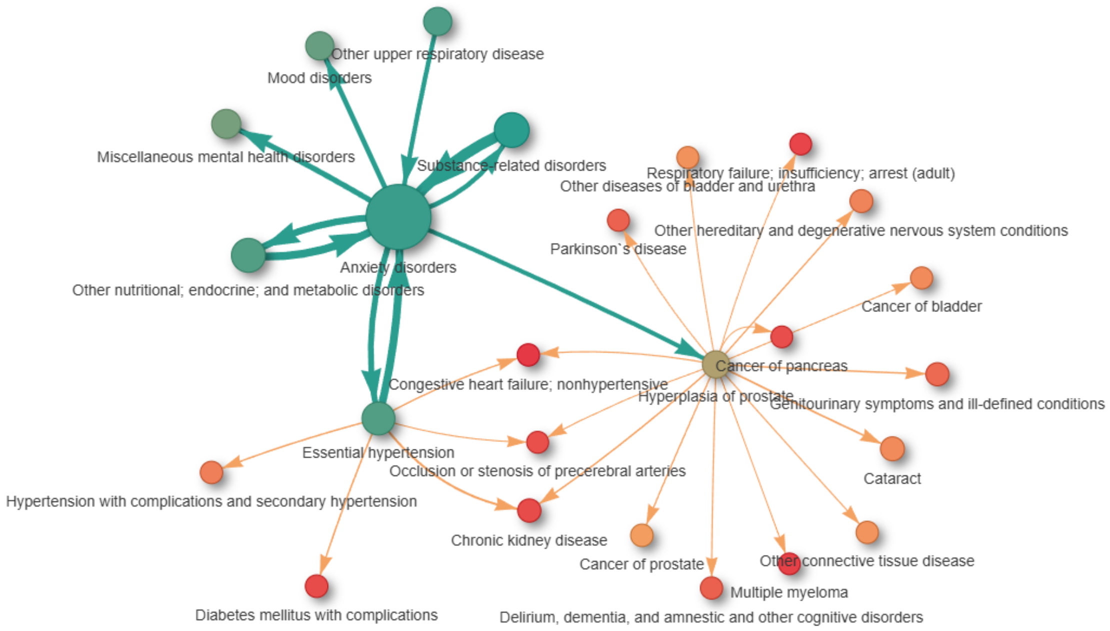

Essential hypertension: Female

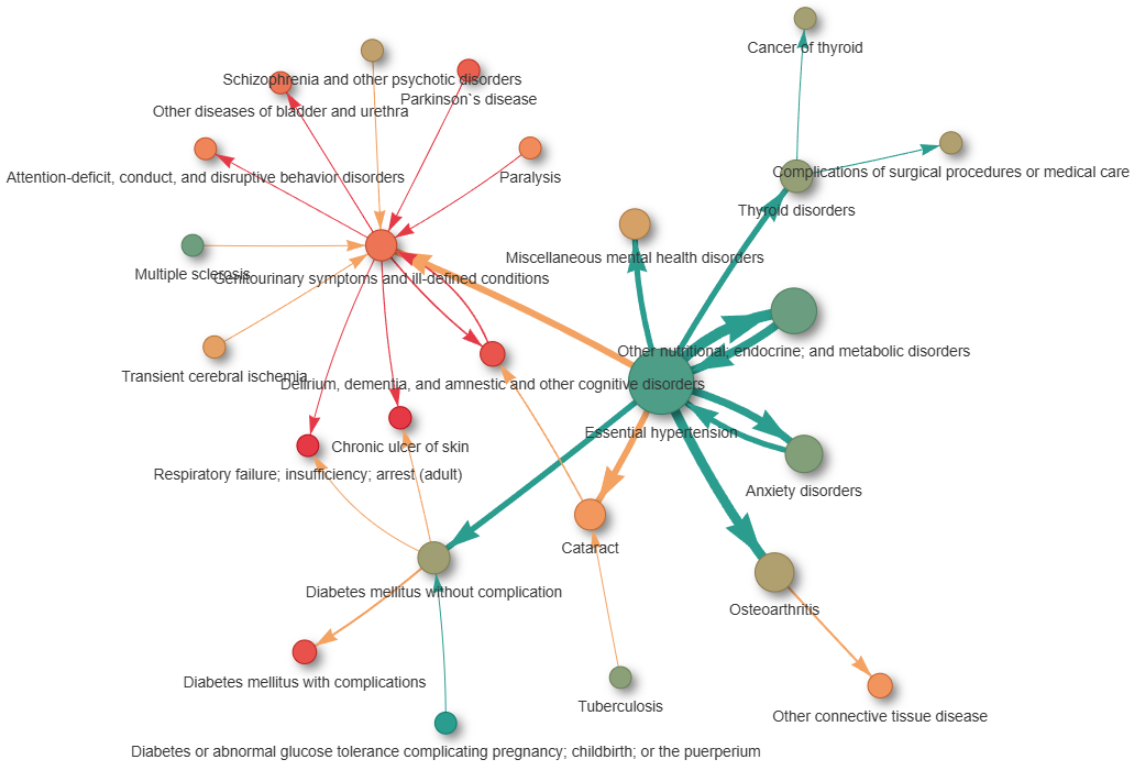

Essential hypertension: Male

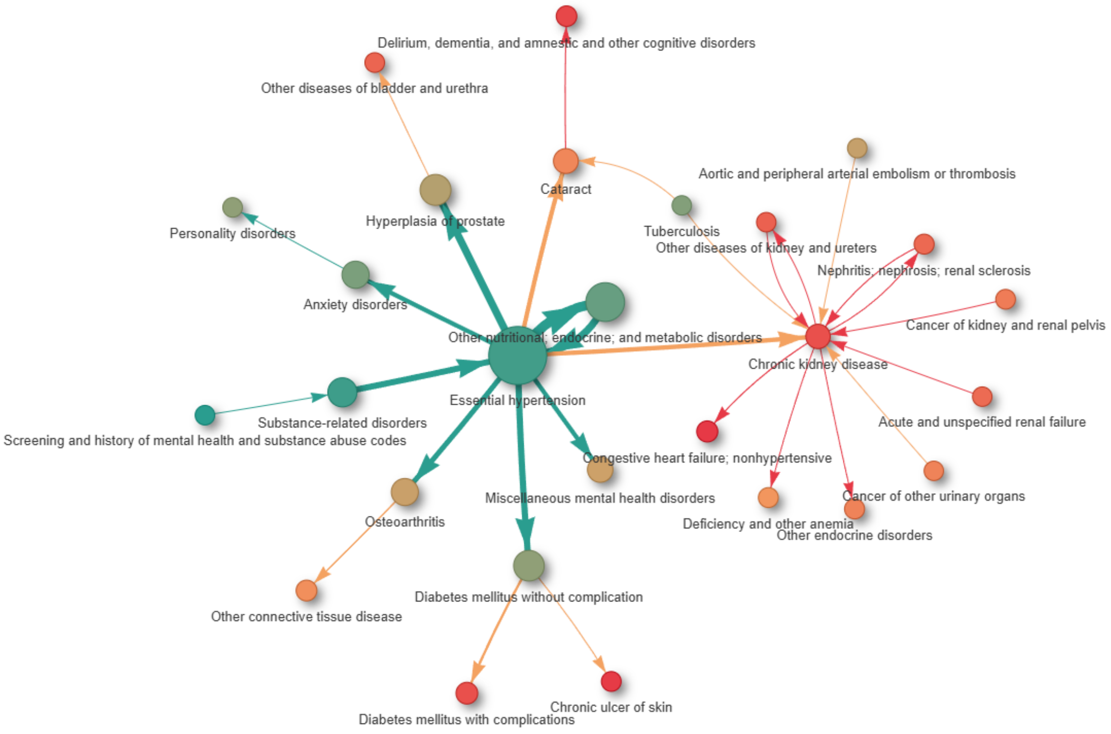

Substance-related disorders: Female

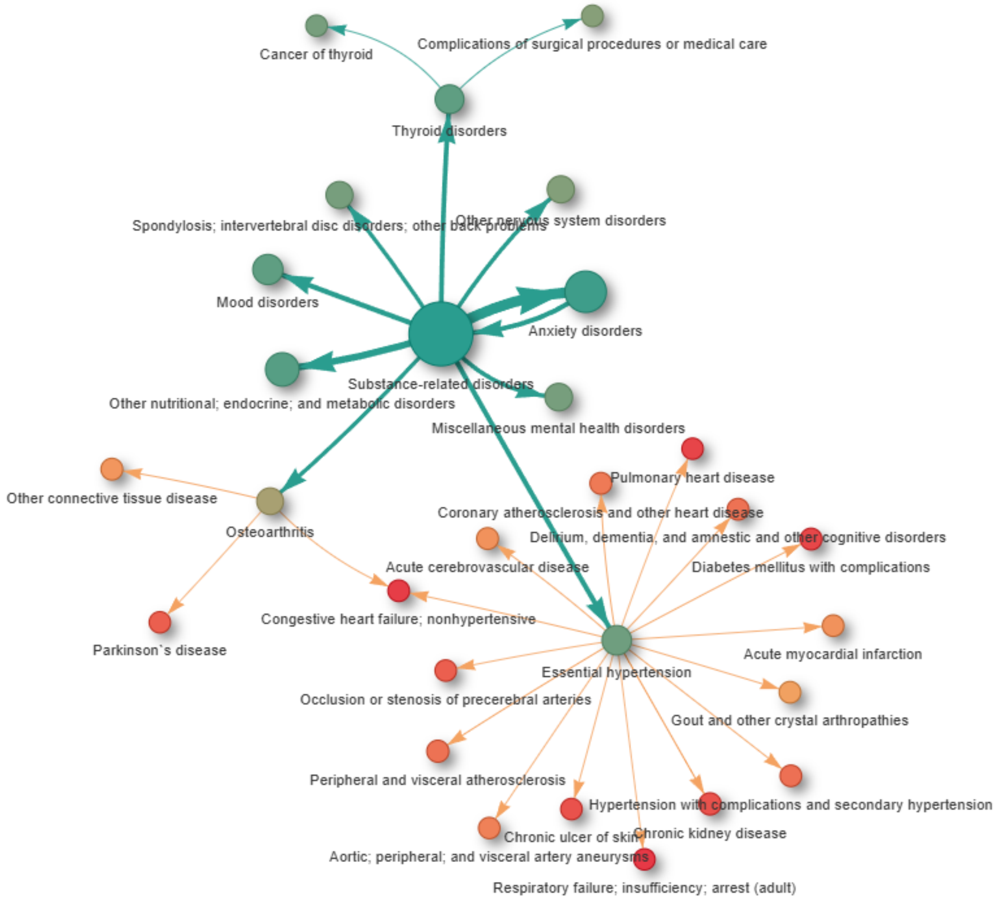

Substance-related disorders: Male

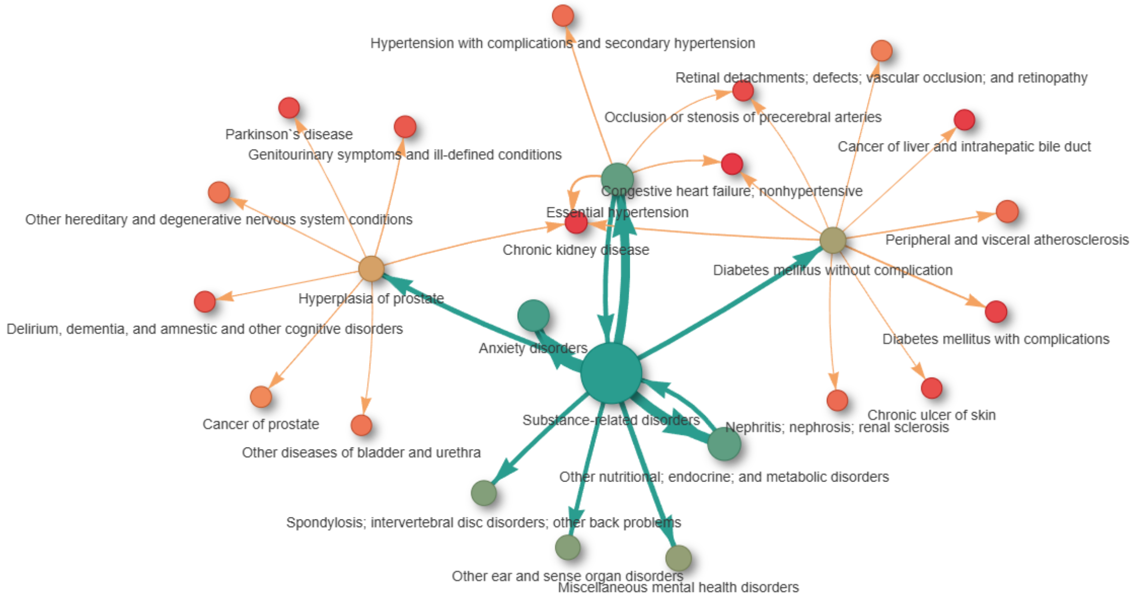

Figure S8. Overview of study design

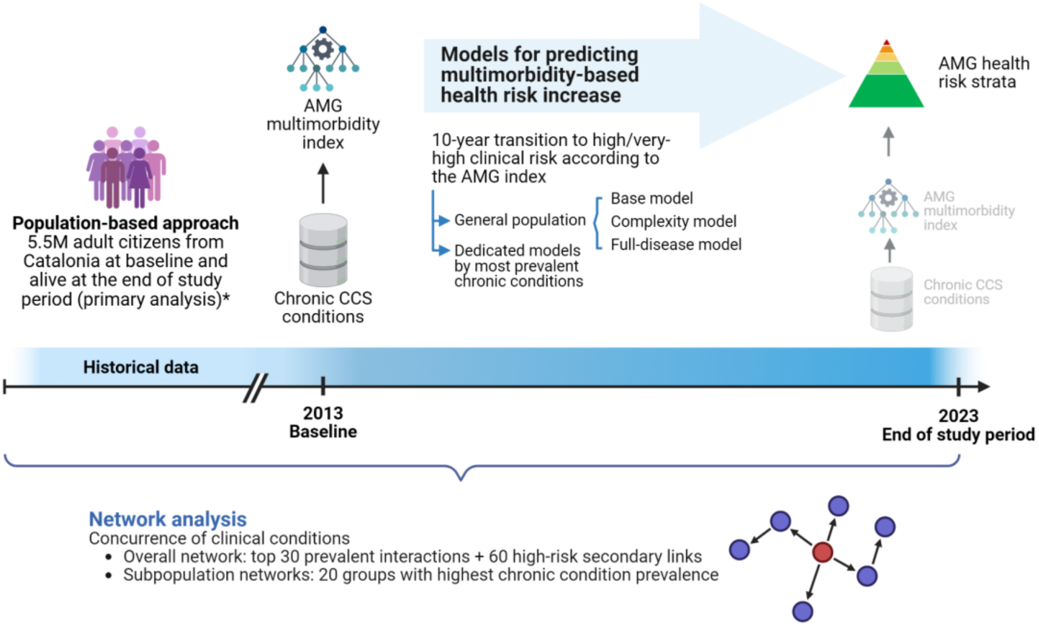

*The primary analysis population consisted of all adult individuals living in Catalonia at the beginning of study period (January 01, 2013) and who were alive by the end of the study period on December 31, 2022. A sensitivity analysis was conducted for individuals who died within the study period.

Figure S9. Processing algorithm of the Adjusted Morbidity Groups

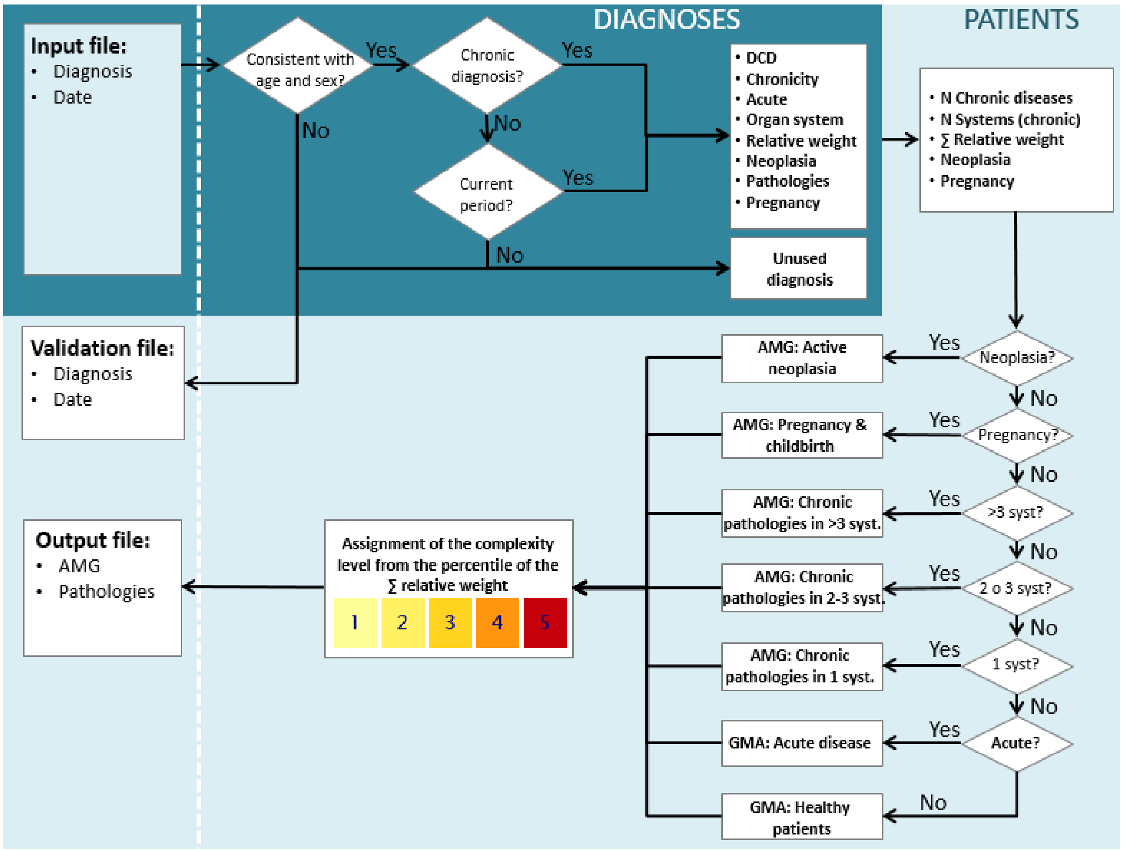

TRIPOD Checklist for Prediction Model Development

Section/Topic	ı	Checklist Item	Page
**Title and abstract**			
Title	1	Identify the study as developing and/or validating a multivariable prediction model, the target population, and the outcome to be predicted.	1
Abstract	2	Provide a summary of objectives, study design, setting, participants, sample size, predictors, outcome, statistical analysis, results, and conclusions.	2
**Introduction**			
Background and objectives	3a	Explain the medical context (including whether diagnostic or prognostic) and rationale for developing or validating the multivariable prediction model, including references to existing models.	3
	3b	Specify the objectives, including whether the study describes the development or validation of the model or both.	3
**Methods**			
Source of data	4a	Describe the study design or source of data (e.g., randomized trial, cohort, or registry data), separately for the development and validation data sets, if applicable.	11
	4b	Specify the key study dates, including start of accrual; end of accrual; and, if applicable, end of follow-up.	11
Participants	5a	Specify key elements of the study setting (e.g., primary care, secondary care, general population) including number and location of centres.	11
	5b	Describe eligibility criteria for participants.	11
	5c	Give details of treatments received, if relevant.	11-15
Outcome	6a	Clearly define the outcome that is predicted by the prediction model, including how and when assessed.	12-13
	6b	Report any actions to blind assessment of the outcome to be predicted.	12-13
Predictors	7a	Clearly define all predictors used in developing or validating the multivariable prediction model, including how and when they were measured.	12-13
	7b	Report any actions to blind assessment of predictors for the outcome and other predictors.	12-13
Sample size	8	Explain how the study size was arrived at.	11
Missing data	9	Describe how missing data were handled (e.g., complete-case analysis, single imputation, multiple imputation) with details of any imputation method.	NA
Statistical analysis methods	10a	Describe how predictors were handled in the analyses.	12-13
	10b	Specify type of model, all model-building procedures (including any predictor selection), and method for internal validation.	13
	10d	Specify all measures used to assess model performance and, if relevant, to compare multiple models.	13
Risk groups	11	Provide details on how risk groups were created, if done.	12
**Results**			
Participants	13a	Describe the flow of participants through the study, including the number of participants with and without the outcome and, if applicable, a summary of the follow-up time. A diagram may be helpful.	4
	13b	Describe the characteristics of the participants (basic demographics, clinical features, available predictors), including the number of participants with missing data for predictors and outcome.	4
Model development	14a	Specify the number of participants and outcome events in each analysis.	4
	14b	If done, report the unadjusted association between each candidate predictor and outcome.	NA
Model specification	15a	Present the full prediction model to allow predictions for individuals (i.e., all regression coefficients, and model intercept or baseline survival at a given time point).	16
	15b	Explain how to the use the prediction model.	12-13
Model performance	16	Report performance measures (with CIs) for the prediction model.	5
**Discussion**			
Limitations	18	Discuss any limitations of the study (such as nonrepresentative sample, few events per predictor, missing data).	10
Interpretation	19b	Give an overall interpretation of the results, considering objectives, limitations, and results from similar studies, and other relevant evidence.	7-9
Implications	20	Discuss the potential clinical use of the model and implications for future research.	7-9
**Other information**			
Supplementary information	21	Provide information about the availability of supplementary resources, such as study protocol, Web calculator, and data sets.	5
Funding	22	Give the source of funding and the role of the funders for the present study.	5
